# Preparation, Supramolecular Organization, and On-Surface Reactivity of Enantiopure Subphthalocyanines: From Bulk to 2D-Polymerization

**DOI:** 10.1021/jacs.2c06377

**Published:** 2022-09-02

**Authors:** Jorge Labella, Giulia Lavarda, Leyre Hernández-López, Fernando Aguilar-Galindo, Sergio Díaz-Tendero, Jorge Lobo-Checa, Tomás Torres

**Affiliations:** † Departamento de Química Orgánica, Universidad Autónoma de Madrid, Madrid 28049, Spain; ‡ Instituto de Nanociencia y Materiales de Aragón (INMA), CSIC−Universidad de Zaragoza, Zaragoza 50009, Spain; § Departamento de Física de la Materia Condensada, Universidad de Zaragoza, Zaragoza 50009, Spain; ∥ Donostia International Physics Center (DIPC), Donostia-San Sebastián 20018, Spain; ⊥ Departamento de Química, Universidad Autónoma de Madrid, Madrid 28049, Spain; # Condensed Matter Physics Center (IFIMAC), Universidad Autónoma de Madrid, Madrid 28049, Spain; ∇ Institute for Advanced Research in Chemical Sciences (IAdChem), Universidad Autónoma de Madrid, Madrid 28049, Spain; ⊗ IMDEA Nanociencia, Campus de Cantoblanco, Madrid 28049, Spain

## Abstract

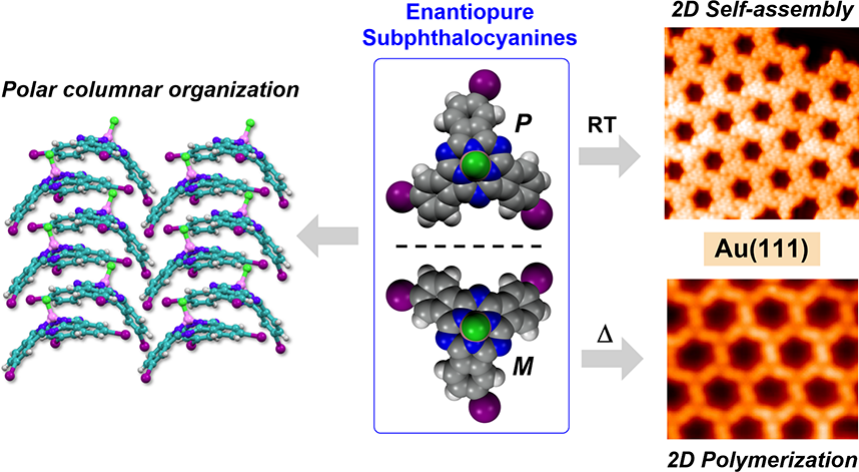

The development of chiral materials is severely limited by the challenge to achieve enantiopure derivatives with both configurational stability and good optoelectronic properties. Herein we demonstrate that enantiopure subphthalocyanines (SubPcs) fulfill such demanding requirements and bear the prospect of becoming components of chiral technologies. Particularly, we describe the synthesis of enantiopure SubPcs and assess the impact of chirality on aspects as fundamental as the supramolecular organization, the behavior in contact with metallic surfaces, and the on-surface reactivity and polymerization. We find that enantiopure SubPcs remarkably tend to organize in columnar polar assemblies at the solid state and highly ordered chiral superstructures on Au(111) surfaces. At the metal interface, such SubPcs are singled out by scanning tunneling microscopy. DFT calculations suggest that SubPcs undergo a bowl-to-bowl inversion that was shown to be dependent on the axial substituent. Finally, we polymerize by means of on-surface synthesis a highly regular 2D, porous and chiral, π-extended polymer that paves the way to future nanodevice fabrication.

## Introduction

Chirality continues to be a fascinating symmetry property with a central role in science.^1^ The inherent chirality of nature identifies it as a critical aspect for biologically focused application areas, such as drug discovery or chemical biology.^2^ Likewise, other fields, such as asymmetric chemical synthesis^3^ or catalysis,^4^ have also extensively investigated the effect of these geometrical attributes. However, only very recently chirality has drawn attention in the rapidly growing field of molecular materials.^5^ In this context, chirality opens a new dimension in the design of novel functional materials, as it provides superb unprecedented molecular properties such as chiroptical responses,^6^ spin selectivity,^7^ or an improved supramolecular organization.^8^ Notably, most state-of-art technological applications (e.g., organic photovoltaics, light-emitting materials, or molecular machines) often require π-conjugated molecules, as they involve light absorption in the visible range and/or semiconducting properties. However, introducing chirality in such systems is highly challenging and far from being trivial.

**Figure 1:**
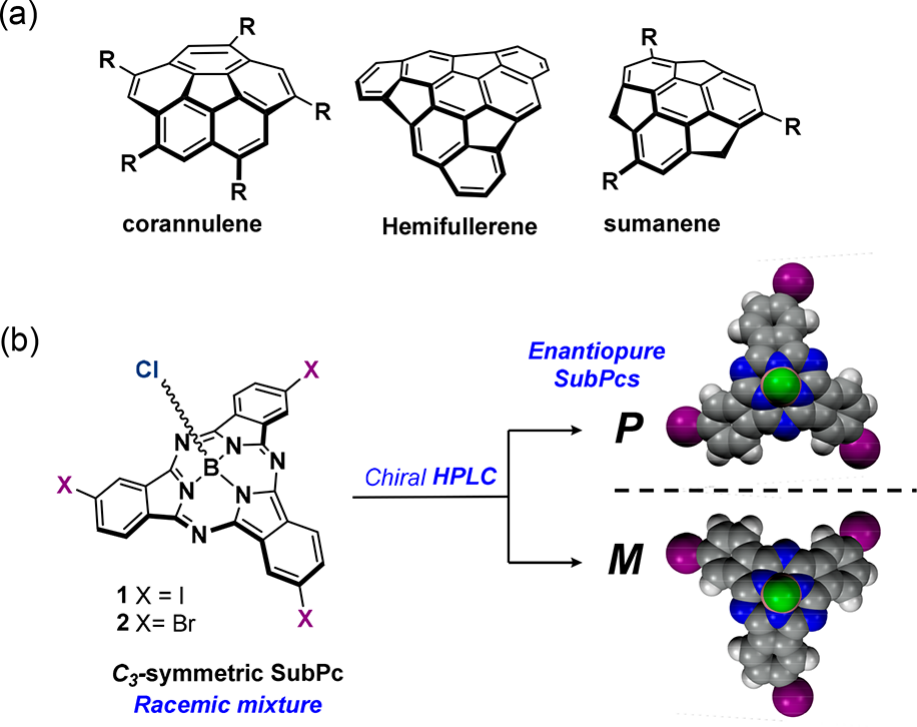
(a) Representative examples of bowl-chiral molecules. (b) Molecular structure of C_3_-symmetric SubPcs and the corresponding *M* and *P* enantiomers. Atom color code: carbon in gray, nitrogen in blue, peripheral halogen atom in purple, axial chlorine in green, and hydrogen in white.

Inspired by fullerene derivatives, an elegant way to achieve chirality in π-systems involves bowl-shaped structures (Figure 1a).^9^ These curved π-systems provide a promising route to fabricate multifunctional devices^10^ since they incorporate additional properties to the material in the form of permanent dipole moments^11^ or columnar arrangements.^12^ Among them, corannulene, sumanene, or hemifullerenes derivatives (Figure 1a) have been most explored due to their exciting electronic properties.^13,14^ However, major drawbacks in the form of poor light absorption profiles (ranging in the near UV) or low energy barriers for the bowl-to-bowl enantiomer conversion prevent the practical use of these derivatives in chiral molecule-based devices. Thus, new guidelines for the design and procurement of chiral compounds, which comprise a correct balance between efficient optoelectronic properties and configurational stability, must be established for the development of novel functional materials.

Subphthalocyanines (SubPcs) (Figure 1b) are excellent candidates to overcome these material limitations. These well-known cone-shaped chromophores consist of three 1,3-diiminoisoindole units assembled around a boron atom.^15^ Their 14π-electron aromatic core and tetrahedral geometry provide outstanding physical and optoelectronic properties in the form of strong dipole moments, excellent light absorption in the 550–650 nm range, rich redox features, and excellent charge transport capabilities. Indeed, SubPcs have already been skillfully exploited in multiple applied fields, such as photovoltaics,^16−18^ nonlinear optics,^19,20^ or organic optoelectronics.^21,22^ Importantly, intrinsic chirality is at reach in SubPcs when prepared by cyclotrimerization of a phthalonitrile with no *C*_2v_ symmetry, and the corresponding enantiomers are stable and can be isolated.^23,24^ Following in-solution preparation of homochiral columnar supramolecular polymers,^25,26^ enantiopure SubPcs are foreseen as exceptional candidates for the production of chiral materials. However, the minute amounts of chiral SubPc obtained so far prevent direct application.^27^ Therefore, an efficient method to produce larger amounts of functionalizable enantiopure samples, as well as their in-depth characterization, is an essential step for the understanding, development, and applicability of chiral SubPcs.

Herein we overcome such material production limitations and report the preparative-scale optical resolution of racemic *C*_*3*_-symmetric SubPc bearing functionalizable peripheral bromine and iodine atoms (**1**–**2**; Figure 1b). We further assess the impact of chirality in the arrangement both on the solid-state (bulk crystal) and in contact with a metal interface. Specifically, we first study the differences in the solid-state arrangement between racemic and enantiopure samples by X-ray diffraction (XRD) analysis. We find that, in contrast to the racemic mixtures (*Rac***1**–**2**), the pure enantiomers of **1**–**2** tend to stack in polar columnar structures. Contrarily, at a metal interface we find that, despite using distinct *M* and *P* enantiomers of **2** (*M***2** and *P***2**), two opposite highly ordered chiral SubPc-based lattices are observed on Au(111), as extracted from low-temperature Scanning Tunneling Microscopy (STM) measurements. Density Functional Theory (DFT) calculations shed light into such unexpected results and conclude that SubPc molecules can undergo a surface-catalyzed dechlorination followed by a bowl-to-bowl inversion that perfectly explains the chiral configuration mixture. Remarkably, such a racemization process can be suppressed by functionalizing the SubPc with an axial fluorine atom (*M* and *P* enantiomers of SubPc **3**, *M***3** and *P***3**). Finally, we exploit the symmetry and on-surface stability of *M***3** and prepare an unprecedented porous, chiral π-conjugated polymer reminiscent of a honeycomb-type lattice via on-surface Ullman-coupling polymerization.

## Results and Discussion

### Synthesis and Resolution of Chiral SubPc 1–2

**Figure 2:**
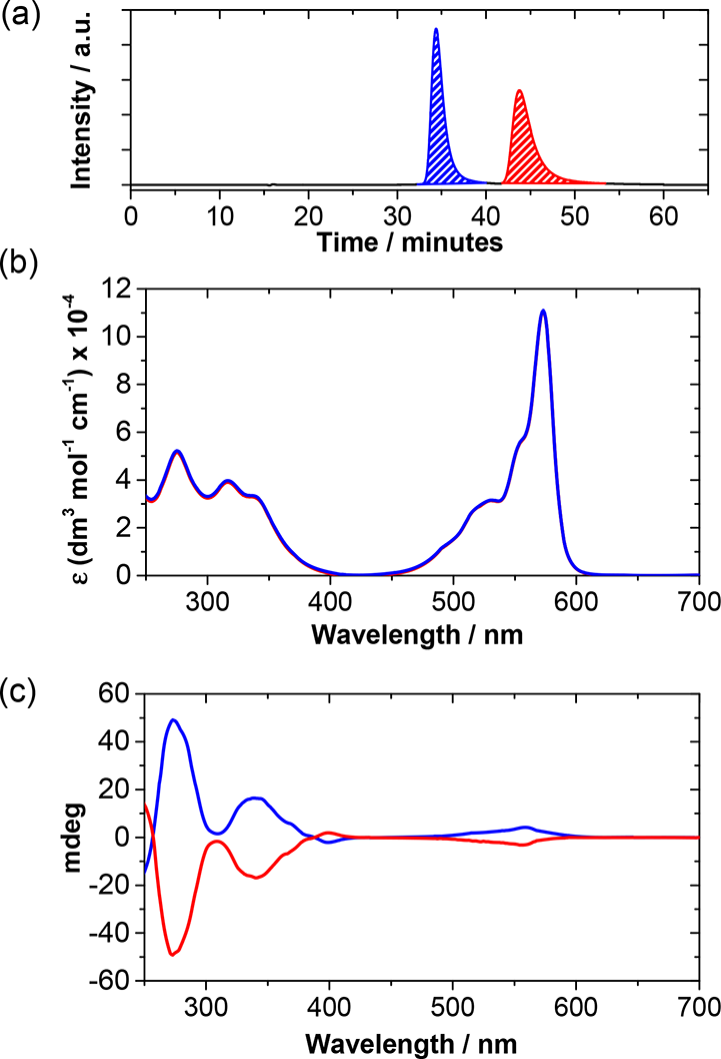
(a) HPLC chromatogram of triiodo-SubPc racemate **1** with peaks corresponding to enantiomers *M* (blue trace) and *P* (red trace). (b) UV–vis absorption spectra of enantiopure SubPcs *M***1** (blue spectrum) and *P***1** (red spectrum) in CHCl_3_ (concentration = 7 × 10^–6^ M). Note that red and blue lines overlap. (c) Circular dichroism spectra of enantiopure SubPcs *M***1** (blue spectrum) and *P***1** (red spectrum) in CHCl_3_ (concentration = 2 × 10^–5^ M). These graphs evidence the excellent enantiopurity of the enantiomers.

*C*_3_-symmetric SubPcs **1**–**2** were prepared as racemic mixtures as detailed in the Supporting Information. With these compounds in hand, the two enantiomers of **1**–**2**, hereafter referred to as *M***1**–**2** and *P***1**–**2**, were obtained in a semipreparative scale by chiral resolution performed on an HPLC equipped with a semipreparative chiral stationary phase column (see Section 4 of the Supporting Information for further details). As shown in Figure 2 and the SI, both of the obtained chromatograms of **1**–**2** display two signals with similar integration values. These eluted compounds showed perfect mirror-image Circular Dichroism (CD) spectra with opposite Cotton effects. As suggested by the theoretical CD spectra simulated by time-dependent Density Functional Theory (TD-DFT) calculations (Figure S5.4), the first eluted fractions correspond to the *M* enantiomers of **1**–**2** (*M***1** and *M***2**), while the second ones correspond to the *P* enantiomers (*P***1** and *P***2**). This assignment has been confirmed by XRD analysis (vide infra).

### X-ray Characterization

**Figure 3:**
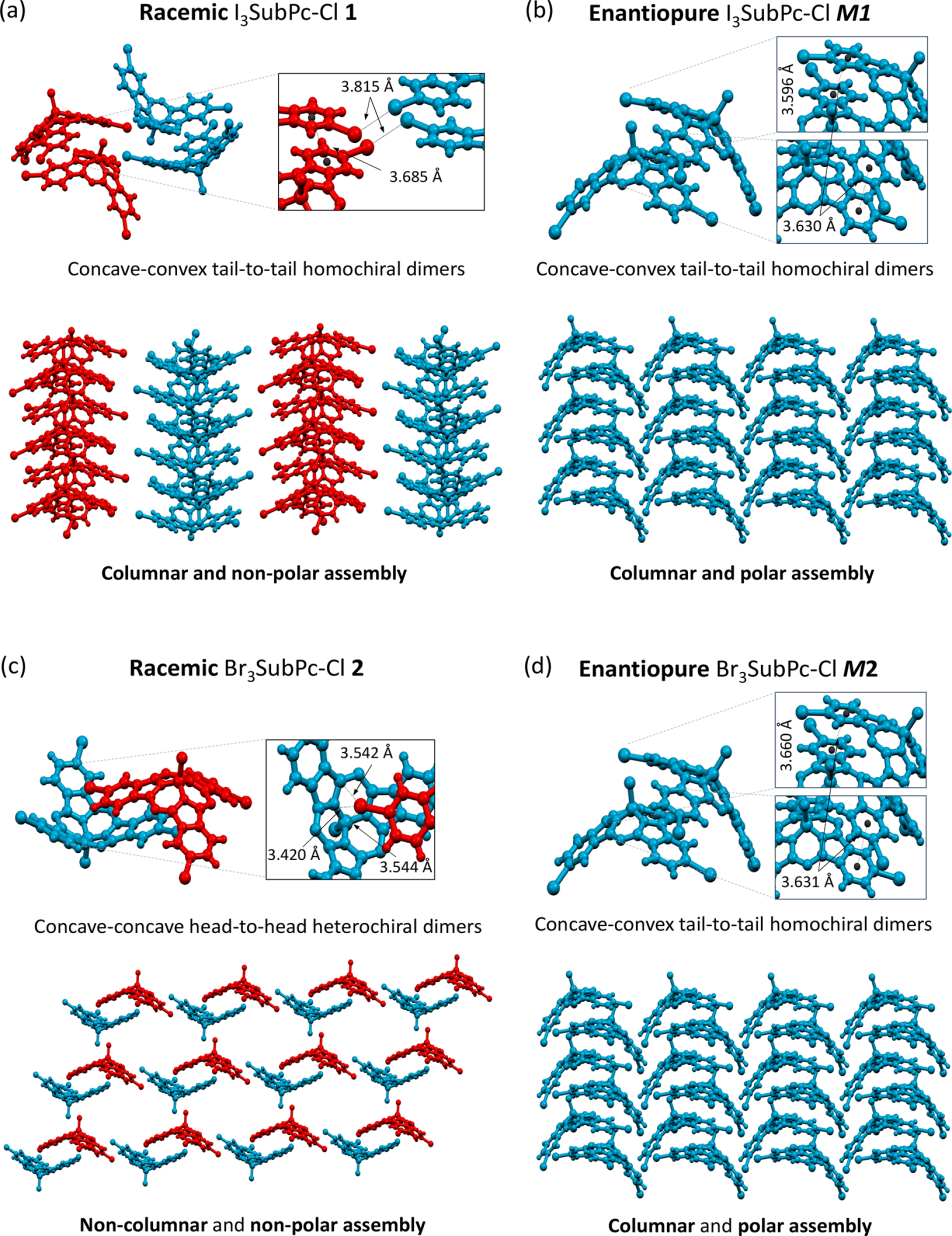
Unit cell (top) and crystal packing (bottom) found in the X-ray structure of (a) *Rac***1**, (b) *M***1**, (c) *Rac***2**, and (d) *M***2**. Red and blue colored molecules correspond to ***P*** and ***M*** enantiomers, respectively. Note that the enantiopure crystals (b) and (d) display almost identical polar structures, whereas the packing in racemic samples (a) and (c) are utterly different.

Aside from characterizing in-depth the single molecules, it is crucial to understand their bulk, collective behavior to design high-performance devices. The solid-state organization controls fundamental aspects such as the charge transport properties or the compound stability.^28,29^ Hence, assessing the impact of the bowl-shaped chirality in this sense is essential to define the material properties. In this way, we first compare the crystal structures of enantiopure SubPcs with respect to the racemic mixture. Specifically, we perform XRD analysis of single crystals of *Rac***1**–**2** and enantiopure *M***1**–**2** and *P***1**–**2**, which were obtained by slow diffusion of hexane into a CHCl_3_ solution (Figure 3).

Remarkably, we find that the enantiopurity steers the supramolecular organization (Figure 3). The triiodo-SubPc *Rac***1** organized in concave–convex, tail-to-tail homochiral dimers through π–π interactions between the isoindolic benzene rings. In turn, these dimers are arranged in homochiral columnar stacks. Within the columns, stabilizing Cl−π interactions between the axial halogen atom and the isoindolic benzene ring of molecules belonging to adjacent stacked dimers are noticeable. While in one direction of the crystal, alternating homochiral columns formed by molecules of opposite chirality are organized in an antiparallel arrangement, in another direction columnar stacks of the same chirality run parallel between them (see Figure S6.3–4). Overall, the racemic species of **1** exhibits a columnar solid-state organization with no net polar moment (Figure 3a). Similar to the racemic, the enantiopure species *M***1** and *P***1** are arranged in concave–convex, tail-to-tail homochiral dimers which show identical stabilizing interactions and columnar assemblies. However, in this case the columnar structures are oriented parallel, giving rise to highly directional polar assemblies (Figure 3b).

On the other hand, *Rac***2** are organized in concave–concave head-to-head (cv–cv h–h) dimers^15^ linked together (Figure 3a).^30^ Such dimers interact with the neighboring dimers by means of Br···N_im_, π–π, or B–Cl···π_pyr_ interactions, resulting in a packing with no directional preference. By contrast, the pure enantiomers *M***2** and *P***2** arrange in a concave–convex tail-to-tail similar to those of obtained with *M***1** and *P***1**, and which are likewise oriented parallel. Therefore, *M***2** and *P***2** also yield columnar and polar solid-state organization (see Figure 3d).

It is noteworthy that the polar organization of *M***1**–**2** and *P***1**–**2** are expected to be less stable than the antiparallel orientation, which are the most commonly observed for bowl-shaped molecules due to the presence of dipole–dipole cancellation.^31,32^ Indeed, our results are in stark contrast to those reported by Miyajima and co-workers, who found that the β-substituted SubPcs tend to form nonpolar crystals.^33^ Thus, this work represents a paradigmatic example of how chirality, assisted by intermolecular interactions, can enable a switching between polar and nonpolar solid-state organization. In the case of *M***1** and *P***1**, a combination of I–I and I−π intercolumnar interactions offset the energy gained in the polar orientation. In the case of *M***2** and *P***2**, Br···π and C–H···N_im_ interactions are responsible for such unexpected stabilization. These singular polar assemblies render enantiopure SubPcs extremely intriguing for potential applications as more efficient semiconductors and materials with ferroelectric behavior and bulk photovoltaic effect (BPVE).

### On-Surface Supramolecular Organization at the Metal Interface Using STM

Such bulk ordering becomes generally disturbed at the interface when contacted with metal leads (electrodes), which are ubiquitous components of molecular devices. At the molecule–metal interface, the organic material can adopt many different configurations, exhibit multiple supramolecular organizations, or undergo a variety of surface-assisted chemical reactions. All these aspects have an enormous impact on the ultimate performance of the fabricated device. Thus, we test the order and interactions of our enantiopure SubPcs when in contact to the three (111) noble metal surfaces. We do so by evaporating racemic and enantiopure SubPcs at room temperature on these substrates and study their arrangement by means of constant current, low temperature (5K) STM imaging, which can exceptionally provide single-molecule resolution.^34,35^ As exemplified in the case of *Rac***1** and *M***1** (see Figure S7.1) we find that Ag(111) and Cu(111) yield irregular structures which were difficult to scan. Such results can be ascribed to the higher reactivity of these surfaces compared to the Au(111) surface,^36,37^ and also to the spontaneous C–I, C–Br, and B–Cl bond cleavage upon arrival at the room temperature surface. To prevent such SubPc alteration we focus on the less reactive surface of the three, the Au(111). Even for the weakest C–I bond, we find that the synthesized SubPcs apparently maintain their integrity when deposited onto Au (see Figure S7.2) leading to better ordered structures compared to Ag or Cu.

**Figure 4:**
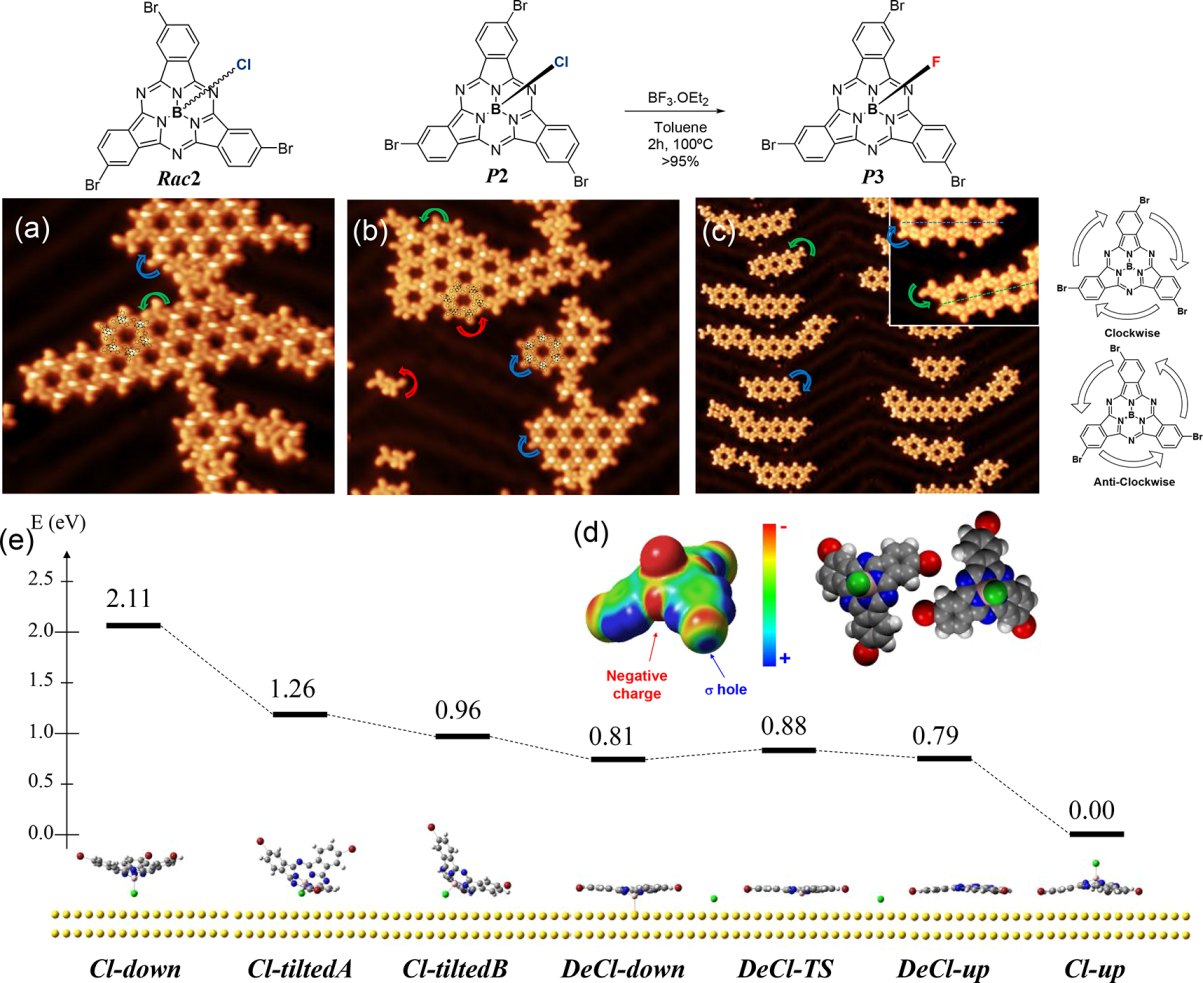
Adsorption and chirality determination of different SubPcs on Au(111) studied by molecular resolved STM imaging: (a) *Rac***2**, (b) *P***2**, and (c) *P***3**. A generalized bowl-down configuration and two chiralities (residual for *P***3**) are found independently of using racemic or enantiopure compounds. The color arrows indicate the “propeller” rotation direction of the three peripheral halogen atoms. The red arrows in (b) indicate the chirality of molecules that have lost their axial dipole. The inset in (c) shows the integrity of the B–F axial dipole independently of the rotation, and the discontinuous green and blue lines allow to easily distinguish *type* II molecular islands with respect to *type* I islands (∼13° deviation). STM image details: (a) *V*_bias_ = −1.0 V, *I*_s_ = 60 pA, size = 20 × 20 nm^2^; (b) *V*_bias_ = 0.1 V, *I*_s_ = 260 pA, size = 25 × 25 nm^2^; (c) *V*_bias_ = 0.1 V, *I*_s_ = 250 pA, size = 50 × 50 nm^2^ (inset size = 11 × 11 nm^2^). (d) ESP map of *P***2** simulated by DFT calculations (CAM-B3LYP/6-31G(d)). Atom color code: carbon in gray, nitrogen in blue, bromine atom in red, axial chlorine in green, and hydrogen in white. The simulation localizes a negative charge at the meso nitrogen atoms, while the bromine atoms exhibit a positive charge in the direction of the σ-bond axis. Such an electronic distribution would enable the formation of stabilizing electrostatic interactions between neighboring SubPcs yielding the observed molecular pattern. (e) Energy profile and calculated structures of the DFT reaction mechanism of the on-surface assisted dechlorination and bowl-to-bowl inversion of *M***2**.

The SubPc bowl-shaped symmetry generally presents two different configurations on a surface: the so-called “bowl-down” (central dipole pointing to the surface) or “bowl-up” (central dipole pointing away from the surface).^38−41^ These SubPcs are imaged by high-resolution STM as propeller-shaped structures with clockwise or anticlockwise rotation, which mirrors their top-view chiral adsorption.^42^ Indeed, as exemplified in Figure 4a for the case of the racemic tribromo-SubPc (*Rac***2**) on Au(111) (Figure S7.2 shows identical arrangements for *Rac***1**–**3**), both chiralities are readily identified on the surface (indicated by green (***M***-isomer) and blue (***P***-isomer) arrows). Importantly, each nanoporous island exhibits a defined chirality, where six molecules rotated by 60° delimit each pore. Note that such honeycomb structure is common to SubPcs on Au(111)^43^ and dominates at RT for these three SubPc derivatives (cf. Figure S7.2d–f). However, it is noted that **1** exhibits other denser assemblies on the surface. As the surface adsorption imposes a lateral (planar) interaction between neighboring molecules, the self-assembled islands are found to be stabilized by electrostatic bonds between the peripheral halogen atoms and the core N_im_ atoms (cf. electrostatic potential (ESP) map of Figure 4d).

To define the role of the chirality, we study the arrangement of the enantiomers on Au(111). Unexpectedly, we still identify two types of chiral islands when depositing the *P***2** enantiomer, as shown in Figure 4b (see Figure S7.2 for the other enantiomers). Particularly, we find three kinds of individual molecules: *type* I that are propellers with clockwise rotation and with a central bright spot (following the enantiomer model, blue arrow), *type* II that are identical with the previous but with an opposite (anticlockwise) rotation (green arrow), and *type* III that feature anticlockwise rotation without the central bright spot (red arrow). Out of roughly 1500 molecules their relative proportion is respectively 34.5%, 25.4%, and 40.1%. The bright spot is generally ascribed to the axial substituent,^37−45^ so *type* III molecules could either be assigned to SubPcs that lost their axial chlorine atom or alternatively could be inverted in a “bowl-up” configuration (dipole pointing toward the Au surface). We discard the latter since the relative intensity of their π-skeletons is identical with the other two, suggesting a generalized “bowl-up” configuration that agrees with previous first layer SubPcs adsorption on Au(111).^38^ The highly unexpected finding that our SubPc enantiomers show two chiralities despite presenting a generalized bowl-down configuration on the surface demands further attention.^46,47^

This on-surface catalyzed bowl-to-bowl inversion can be understood by performing quantum chemistry simulations using DFT (see the SI Materials and Methods for further computational details).^48,49^ We thoroughly explored the potential energy surface, searching for minima of *M***2** (identical with *P***2**) conformers adsorbed on a Au(111) surface. Specifically, the relative energy of both bowl-down and bowl-up, with and without axial chlorine atom, were calculated. Hereafter, we referred to them as *Cl-Up*, *Cl-down*, *DeCl-up*, and *DeCl-down*, respectively. Furthermore, we also computed intermediate configurations where the convex face of the SubPc is directly interacting with the surface through one or two isoindolic moieties (*Cl-tiltedA* and *Cl-tiltedB*, respectively). Results are summarized in Figure 4e, and all the considered structures are given in the SI. By inspection of the computed relative energy, it can be concluded the bowl-up configurations that keep the axial chlorine, *Cl-down* (Δ*E* = 2.11 eV), *Cl-tiltedA* (Δ*E*= 1.26 eV), and *Cl-tiltedB* (Δ*E* = 0.96 eV), are significantly less stable than the *Cl-up* structure. Notably, the dechlorinated configurations (*DeCl-up* and *DeCl-down*) are more stable than *Cl-down*, *Cl-tiltedA*, and *Cl-tiltedB*. Therefore, as shown in the reaction pathway depicted in Figure 4e, it is expected that a SubPc adopting a bowl-up configuration (*Cl-down*, *Cl-tiltedA*, or *Cl-tiltedB*) will evolve toward the more stable configuration by cleavage of the axial chlorine atom. Overall, we can draw the following model to explain the experimental results: enantiomer *P***2** can adopt two configurations when reaching the surface, *Cl-up* or *Cl-down*. The *Cl-up* molecules remain intact since they are in the most stable configuration (*type* I), while molecules in the *Cl-down* configuration evolve into *Cl-tiltedA* and *Cl-tiltedB*, which after axial dechlorination follow by a bowl-to-bowl inversion yielding *DeCl-up* (*type* III). Such inversion takes place through the transition state *DeCl-TS*, which shows a low energy barrier (0.07 eV) and leads to a more stable configuration. As residual chlorine atoms can remain on the surface, the axial position of the SubPc can be reoccupied leading to *Cl-Up* (*type* II) molecules. Thus, despite selectively depositing a ***P*** or ***M*** enantiomer, the opposite chirality emerges evolving from dechlorinated Cl-down configurations (*types* II and III). This hypothesis is further supported by the fact that experimentally there are no molecules of *type* I chirality without the central bright spot since the axial chlorine atom never contacts the surface, and thus the dechlorination cannot be initiated. Indeed, we can even estimate the ratio between Cl-up and Cl-down SubPcs when deposited on Au(111): *type* I molecules must land on the surface with the plane formed by their three Brs at any angle smaller than the normal to the surface. This leads to ∼35% of the events according to a simple geometrical estimation of the enclosed spherical cap volume under this plane. The rest of the molecules (∼65%) of the events eventually lead to geometries with the axial ligand pointing to the surface that will result in *type* II and III species.^50^

In light of these results, we exchange the axial chlorine with a fluorine atom to increase the axial stability and prevent the axial dechlorination.^51^ To this end, *M***2** and *P***2** were reacted with BF_3_·OEt_2_ affording the corresponding fluorinated SubPcs *M***3** and *P***3**. It should be stressed that although a residual amount of the opposite enantiomer is noticeable,^52^ the chirality is maintained during this chemical transformation (see Section 4 of the Supporting Information). This result can be easily explained by the bimolecular mechanism found for other axial substitutions on SubPcs.^53^ Note that the retention of configuration confirms the excellent configurational stability of SubPcs, even after undergoing an axial substitution at high temperatures.

The deposition of *P***3** (Figure 4c) and *M***3** (Figure S7.2) on the surface of Au(111) kept at room temperature also reveals the familiar nanoporous honeycomb-like structures. However, the STM images show a clear predominance of *type* I molecules on the surface and only a residual amount of *type* II SubPcs with absence of *type* III molecules (cf. Figure 4c). The B–F axial ligand is more difficult to image than for the previous SubPcs, but with the proper tip termination the central part of the molecules are imaged as bright protrusions (see Figure S7.5). Hence, as shown in the inset of Figure 4c, the superior strength of the B–F avoids the loss of this axial ligand and accordingly minimizes the bowl-to-bowl inversion. This hypothesis is further supported by comparing the calculated energy of the key intermediates F-up, F-down, and DeF-down with the B–Cl axial ligand (see Figure S8.2). Interestingly, the minute amounts of *type* II molecules of *P***3** (or *M***3**) still aggregate into homochiral islands. Thus, the chiral recognition and segregation is a general aspect both in racemic and enantiomer compounds on the surface of Au(111) (see Figure S7.2).^54^ As already suggested from the STM high-resolution images and confirmed by the DFT electrostatic potential map of *M***2**, such hexagonal arrays nucleate by the stabilizing dipole–dipole N_im_···Br and C–H···Br pairs established between homochiral molecules (Figure 4d). In essence, this resembles the previously discussed bulk crystal bonds, but restricted to 2D (lateral) interactions.

### On-Surface Ullman-Coupling Polymerization

The peripheral halogens that introduce the SubPc chirality can be exploited to polymerize the molecules by means of an Ullman coupling processes.^55,56^ Many of such on-surface chemical reactions have enabled the synthesis of complex systems otherwise impossible to prepare in conventional solution (or heterogeneous) chemistry.^57^ In this context 2D π-extended polymers are interesting due to their potential use in optoelectronic devices,^58^ where chirality^59,60^ or porosity^61^ endows intriguing additional properties. To date, the preparation of a 2D π-polymer that combines chirality and porosity into a single framework remains an open challenge.

**Figure 5:**
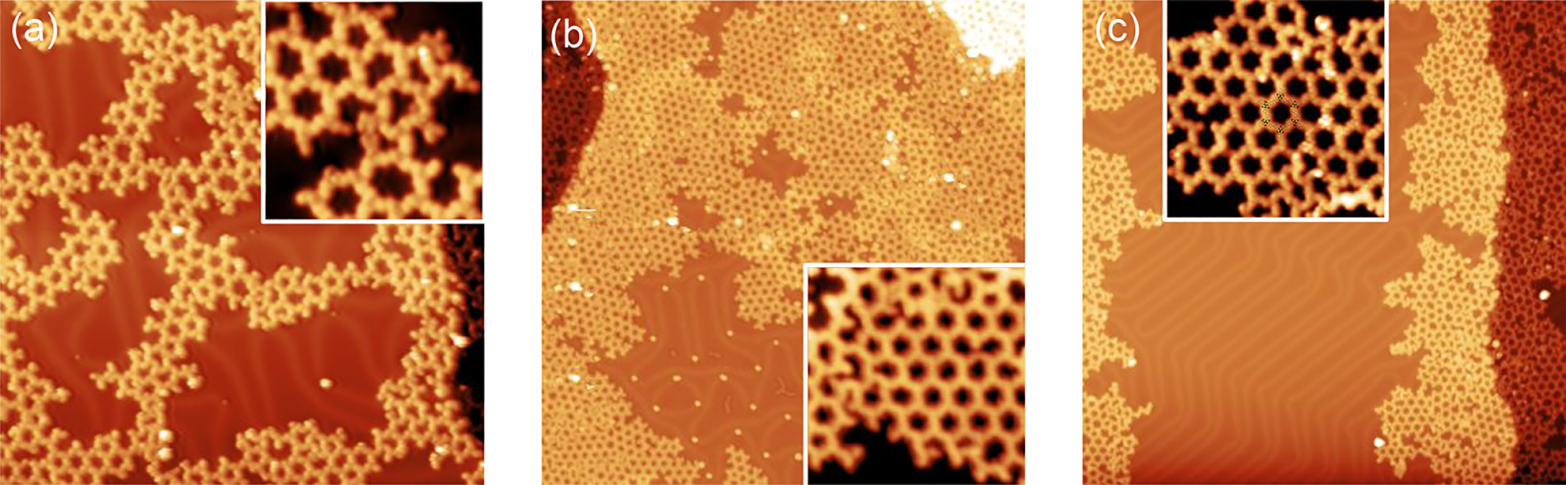
STM images at 5K of the *M***3** after polymerization on the Au(111) surface under different conditions. (a) Sample postannealed to 210 °C after room temperature deposition, (b) low deposition rate (over 90 min) with the substrate kept at 220 °C, and (c) postannealing the latter for 20 min up to 350°. STM image details: (a) *V*_bias_ = −1.0 V, *I*_s_ = 100 pA, size = 50 × 50 nm^2^ (inset: *V*_bias_ = 0.2 V, *I*_s_ = 100 pA, size = 10 × 10 nm^2^); (b) *V*_bias_ = −1.0 V, *I*_s_ = 100 pA, size = 100 × 100 nm^2^ (inset: *V*_bias_ = −1.0 V, *I*_s_ = 100 pA, size = 15 × 15 nm^2^); (c) *V*_bias_ = −1.0 V, *I*_s_ = 100 pA, size = 100 × 100 nm^2^ (inset: *V*_bias_ = −1.0 V, *I*_s_ = 100 pA, size = 15 × 15 nm^2^).

By inspecting the generic molecular structure of enantiopure SubPcs (Figure 1), we envision that these derivatives present ideal symmetry characteristics to overcome such challenges. In this way, we tested the polymerization process to synthesize 2D, porous, and chiral SubPc-based π-polymers. As shown in Figure S7.3, all racemic and enantiomer compounds can be polymerized when annealed above 200 °C on the Au(111) substrate. However, our previous findings position the enantiomers of **3** as the most promising candidates for achieving regular structures given the single kind of molecules (*type* I) that dominate on the surface and also their highest axial stability. The structures obtained with *Rac***3** turn out to be highly disordered due to the intermixing of the two chiral species (cf. Figure S7.3c). Contrarily, when using a SubPc enantiomer, the structures become more regular in comparison (see Figure 5a and Figure S7.3f). Such disorder reduction suggests that the molecules preferentially maintain a bowl-down configuration when covalently bonding (cf. Figure S.7.5e,f). Thus, the well-defined chirality turns out to be an excellent option to generate extended and regular 2D-polymers. Despite this, we find that the *M***3** polymerized structures are rather irregular after postannealing the room temperature deposited molecules (cf. Figure 5a). We attribute this to the thermal loss of order combined with the molecular diffusion obstruction by the cleaved bromine atoms. These halogen residues (adatoms) are visualized as faint spheres surrounding the polymeric structure or in the middle of the terraces. They persist on the surface until approximately 300 °C, well above the dehalogenation onset occurring well below 200 °C.

To overcome such hindering effect of the cleaved Br atoms, we severely change the deposition conditions in two ways: first we use a high substrate deposition temperature (above dehalogenation) and second, we drastically reduce the evaporation rate so that the polymer would completely cover the surface after 4 or 5 h. In this way, the SubPcs arriving to the hot surface spontaneously cleave their Br atoms and are also granted with long enough times to diffuse and find another in kind to covalently bind. As shown in Figure 5b and Figure S7.4a, this process considerably improves the polymer structure regularity, leading to 2D polymers flawlessly extending over regions larger than 100 nm^2^. The deposition temperature used (well below 300 °C) allows one to visualize these bromine adatoms (clearly identified in Figure 5b and Figure S7.4a). The desorption of these adatoms above 300 °C leaves the 2D polymeric structures unperturbed (cf. Figure 5c and Figure S7.4b). Interestingly, at those elevated temperatures the axial B–F ligands are also unmodified (cf. Figure 5c and Figure S7.4b insets). Therefore, we can precisely determine the geometry of a regular 2D nanoporous polymer as made up of six molecules, with the B–F at the hexagonal corners and maintaining the *type* I chirality on their covalent bond (see insets of Figure 5c and Figure S7.4b).

## Conclusions and Outlook

In summary, we demonstrate the synthesis and efficient optical resolution of *C*_3_-symmetric triiodo- and tribromo-SubPcs that can also be sublimated in a vacuum. Remarkably, enantiopure derivatives significantly improve the supramolecular organization in comparison with the racemic, which is crucial for potential applications. This is simultaneously accompanied by a switching between polar and nonpolar assemblies at the solid-state when moving from racemic to enantiopure samples, as revealed by X-ray diffraction analysis. Further insight into the self-assembly behavior at the metal–organic interface is provided by STM, where we reveal that all these enantiomers deposited on Au(111) surface organize in highly ordered porous monolayers separated by chirality. Importantly, the opposite enantiomer chirality is also detected due to a bowl-to-bowl inversion induced by the metallic surface. Assisted by DFT calculations, we show that the probability of this inversion is dependent on the stability of the axial substituent, which is minimized as it becomes stronger.

Finally, given the symmetry, stability, and functionalization of the enantiopure compounds of **3**, we generate by Ullman coupling an unprecedented 2D π-polymer which exhibits both chirality and porosity. We find a preferential bowl-down to bowl-down interaction when forming the covalent bonds that is key to improving the regularity of the 2D-polymers. This is further improved by increasing the deposition time and temperature of the substrate to grant sufficient diffusion time to the molecules to recombine.

This work establishes very valuable concepts not only within unexplored aspects of SubPcs (i.e., chirality, on-surface organization/reactivity), but also in the fields of bowl-shaped molecules, polar assemblies, and on-surface synthesis of 2D materials. The enantiopure SubPcs are expected to be key building blocks for next generation chiral materials.

## Supporting Information Available

General information, experimental procedures, new compound characterization, crystallographic data, HPLC chromatograms, NMR spectra, DFT calculations, and STM data (PDF)
