# Supplementary material for: Preparation, Supramolecular Organization, and On-Surface Reactivity of Enantiopure Subphthalocyanines: From Bulk to 2D-Polymerization
Source: J Am Chem Soc. 2022 Sep 2;144(36):16579–87. doi: 10.1021/jacs.2c06377 (PMC9479063; doi:10.1021/jacs.2c06377)
Supplement: Supplementary file 1 — ja2c06377_si_001.pdf [file ja2c06377_si_001.pdf]

Supplementary Information

Preparation, supramolecular organization, and on-surface reactivity of enantiopure Subphthalocyanines: from bulk to 2D-polymerization

Jorge Labella,[a] Giulia Lavarda,[a+] Leyre Hernández-López,[b,c,+] Fernando Aguilar-Galindo,[d,e] Sergio Díaz-Tendero,[e,f,g] Jorge Lobo-Checa,[b,c,*] Tomás Torres[a,g,h*]

[a] Departamento de Química Orgánica, Universidad Autónoma de Madrid, Madrid, 28049, Spain.

[b] Instituto de Nanociencia y Materiales de Aragón (INMA), CSIC-Universidad de Zaragoza, Zaragoza 50009, Spain.

[c] Departamento de Física de la Materia Condensada, Universidad de Zaragoza, E-50009 Zaragoza, Spain

[d] Donostia International Physics Center (DIPC), Donostia-San Sebastián E-20018, Spain.

[e] Departamento de Química, Universidad Autónoma de Madrid, 28049 Madrid, Spain.

[f] Condensed Matter Physics Center (IFIMAC), Universidad Autónoma de Madrid, 28049 Madrid, Spain

[g] Institute for Advanced Research in Chemical Sciences (IAdChem), Universidad Autónoma de Madrid, Madrid, 28049, Spain.

[h] IMDEA-Nanociencia, Campus de Cantoblanco, Madrid, 28049, Spain.

[+] These authors contributed equally to this work.

Table of content

1. Materials and methods S2
2. Synthesis and characterization of SubPcs **2-3** S3
3. Analysis of intrinsic chirality of SubPcs **1-3** S6
4. Semi-preparative HPLC resolution of racemic SubPcs **1-2** and analysis of **3** S6
5. UV-vis, circular dichroism and fluorescence spectra of enantiopure SubPcs **1-3** S8
6. X-ray crystal structure of racemic SubPcs **1-2** and enantiopure SubPcs ***M*1-2** and ***P*1-2** S11
7. On-surface organization and polymerization of racemic SubPcs **1-3** and SubPcs ***M*1-3** and ***P*1-3** S19
8. Theoretical calculations. S22

Abbreviations:

CD = circular dichroism; HPLC = high performance liquid chromatography; HR-MS = high resolution mass spectrometry; MALDI-TOF = matrix-assisted laser desorption/ionization-time of flight; MS = mass spectrometry; NMR = nuclear magnetic resonance; ppm = part per million; SubPc = subphthalocyanine; TLC = thin layer chromatography; UV-vis = ultraviolet-visible; THF = tetrahydrofuran; Mp= melting point; DCTB = *trans*-2-[3-(4-*tert*-Butylphenyl)-2-methyl-2-propenylidene]malononitrile.

1. Materials and methods

Chemicals were purchased from commercial suppliers and used without further purification unless otherwise stated. C_3-I_3SubPcCl (**1**), obtained as a racemic mixture of the *M* and *P* enantiomers (*vide infra*), was prepared according to a previously reported synthetic procedure.[1] Thin layer chromatography (TLC), was carried out employing aluminium sheets coated with silica gel type 60 F254 (0.2 mm thick, E. Merck). Purification and separation of the C_1 and C_3 regioisomers of the synthesized products was performed by column chromatography using silica gel (230–400 mesh, 0.040–0.063 mm, Merck). Matrix Assisted Laser Desorption/ Ionization-Time of Flight (MALDI-TOF) spectra were obtained using a Bruker Ultraflex III spectrometer equipped with a Nd-YAG laser operating at 337 nm. High Resolution Mass Spectrometry (HR-MS) spectra were recorded employing ESI Positive Q-TOF using a Bruker Maxis II. The different matrixes employed are indicated for each spectrum. Mass spectrometry data are expressed in m/z units. ^{1}H-NMR spectra were recorded on a Bruker AC-300 (300 MHz) at room temperature. In the ^{1}H-NMR spectra, the chemical shifts (δ) are measured in ppm relative to the correspondent deuterated solvent. UV-Vis spectra were recorded employing a JASCO-V660 spectrophotometer. CD spectra were recorded with a JASCO V-815 equipment. The synthesis of SubPc **1** has been previously reported.[2] Resolution of racemic SubPcs **1-2** was carried out by chiral HPLC using an Agilent 1200 equipment with a semi-preparative Daicel Chiralpak IC column (10 mm ø x 20 mmL). Single crystals of racemates **1-2** and enantiopures ***M*1-2** and ***P*1-2** suitable for X-ray analysis were obtained by slow diffusion of methanol into a chloroform solution of the corresponding SubPc derivative. The X-ray crystal structure analysis of racemic SubPcs **1-2** and enantiopure ***M*1-2** and ***P*1-2** was performed on a Bruker Kappa apparatus at 200 K using two-dimensional detector Apex II with Mo kα radiation (λ = 0.71073 A). The deposition numbers of the resolved crystal structures in the Cambridge Crystallographic Data Centre are the following: **2170077** (**1**), **2170078** (***M*1**), **2170079** (***P*1**), **2170080** (**2**), **2170081** (***M*2**) and **2170082** (***M*2**). These data can be obtained free of charge from The Cambridge Crystallographic Data Centre.

2. Synthesis and characterization of SubPcs 2-3

C$_3$-Br$_3$SubPc-Cl (2)

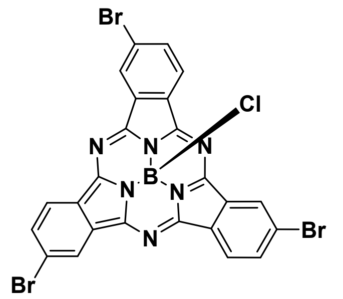

In a 50 mL two-necked round-bottomed flask, equipped with a condenser, magnetic stirrer and rubber seal, a 1.0 M solution of BCl_3 in p-xylene (7.25 mL) was added to 4-bromophthalonitrile (1.5 g, 7.25 mmol) under argon atmosphere. The reaction mixture was stirred at 140 ºC for 20 min. The purple solution was allowed to cool to room temperature and flushed with argon. The dark purple reaction slurry was dissolved in toluene/THF 10:1 and passed through a short silica plug. The solvent was removed by vacuum distillation and the resulting dark solid was purified by column chromatography on silica gel using toluene/heptane 3:1 as eluent. During the process, C_3 and C_1 regioisomers were separated. Upon recrystallization from methanol, 120 mg (0.18 mmol) and 410 mg (0.61 mmol) of C_3 and C_1 regioisomers, respectively, were obtained as purple-gold solids. Yield: 33%. **Mp** > 250 °C; **^{1}H-NMR** (300 MHz, $CDCl_3$): δ (ppm) = 9.01 (d, $^4J_{H-H}$ = 1.7 Hz, 3H), 8.72 (d, $^3J_{H-H}$ = 9.7 Hz, 3H), 8.05 (dd, $^3J_{H-H}$ = 9.7 Hz, $^4J_{H-H}$ = 1.7 Hz, 3H); **^{13}C-NMR** (75.5 MHz, $CDCl_3$): δ (ppm) = 150.3, 149.7, 134.2, 132.9, 130.1, 126.1, 125.6, 124.3; **^{11}B-NMR** (160.5 MHz, $CDCl_3$): δ (ppm) = -13.8 (s); MS (MALDI-TOF, DCTB): m/z = 667.9 [M]+. HRLSI-MS: m/z Calcd for [$C_{24}H_9BBr_3ClN_6$]: 667.8178; Found: 667.8195. **UV/vis** ($CHCl_3$): λ_{max} (nm) (log ε) = 562 (5.0), 305 (4.8).

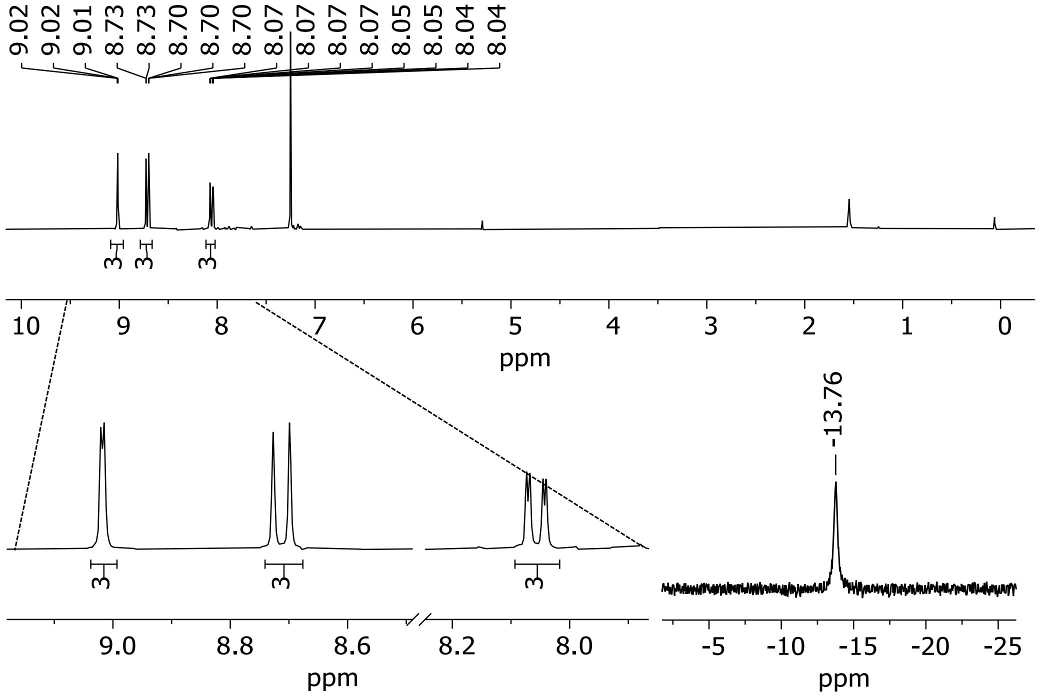

Figure S2.1. ^{1}H- and ^{11}B-NMR spectra ($CDCl_3$) of **2**

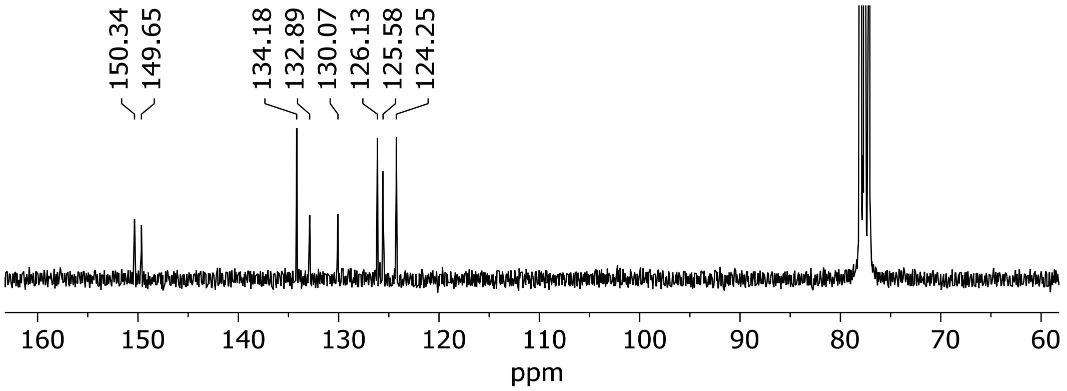

Figure S2.2. ^{13}C-NMR spectrum ($CDCl_3$) of **2**

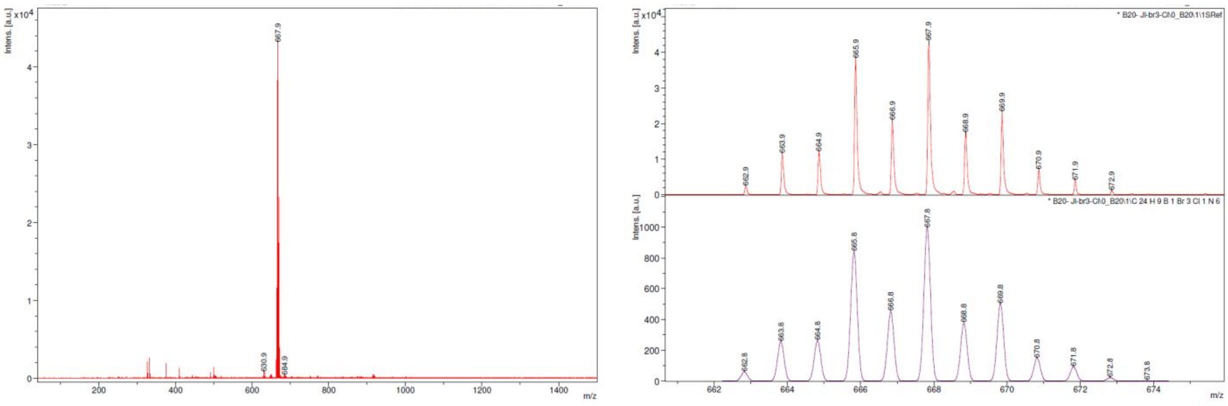

Figure S2.3. MALDI-TOF mass spectrum of **2**.

C_3-Br_3SubPc-F (3)

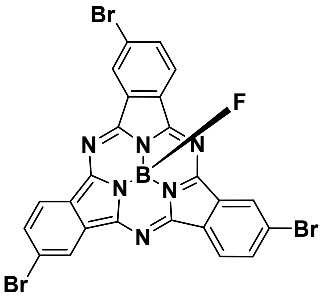

In a 10 mL Schlenk flask, equipped with a magnetic stirrer and rubber seal, **2** (*Rac*, *M* or *P*) 20 mg, 30 μmol) was dissolved in dry toluene (0.5 mL) under argon atmosphere and $Et_2O·BF_3$ (0.18 mL, 1.5 mmol) was added dropwise. The reaction mixture was stirred at 120 ºC for 3 h. The purple solution was allowed to cool to room temperature and pyridine was added dropwise until the color of the reaction returned to a characteristic SubPc pink color. The flask was placed in an ice bath for one hour, and the precipitate subsequently isolated by vacuum filtration. The filter cake was rinsed with methanol followed by ether to give 18 mg (28 μmol) of compound **3** as a purple solid. Yield: 93%; **Mp** > 250 °C. **^{1}H-NMR** (500 MHz, $C_2D_2Cl_4$, 120 °C): δ (ppm) = 9.01 (d, $^{4}J_{H-H}$ = 1.7 Hz, 3H), 8.72 (d, $^{3}J_{H-H}$ = 9.7 Hz, 3H), 8.05 (dd, $^{3}J_{H-H}$ = 9.7 Hz, $^{4}J_{H-H}$ = 1.7 Hz, 3H); **^{13}C-NMR** (500 MHz,

$C_2D_2Cl_4$, 120 °C): δ (ppm) = 151.51, 150.81, 133.74, 132.95, 130.13, 125.83, 125.17, 123.97; **^{19}F-NMR** (470 MHz, $C_2D_2Cl_4$, 120 °C): δ (ppm) = -157.3 (q, J = 28.5 Hz, 1F; B-F); **^{11}B-NMR** (160 MHz, $C_2D_2Cl_4$, 120 °C): δ (ppm) = -14.01 (d, J = 30 Hz, 1B; B-F); MS (MALDI-TOF, DCTB): m/z = 649.9 [M]+. HRLSI-MS: m/z Calcd for [$C_{24}H_9BBr_3FN_6$]: 649.8495; Found: 649.8531. **UV/vis** ($CHCl_3$): λ_{max} (nm) (log ε) = 560 (5.0), 304 (4.8).

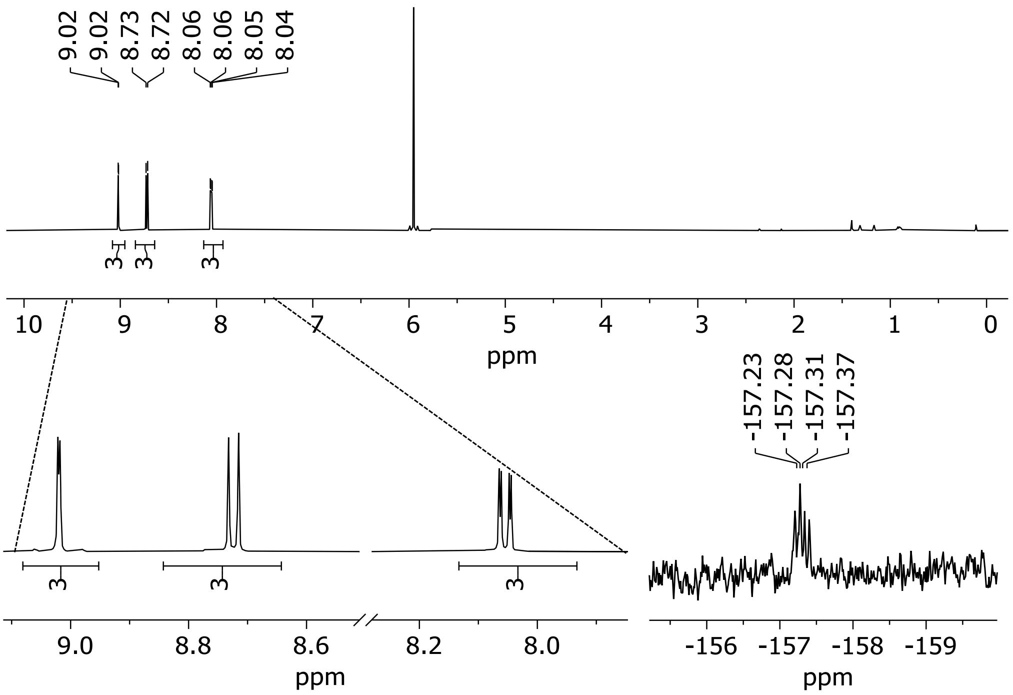

Figure S2.4. ^{1}H- and ^{19}F-NMR spectra ($C_2D_2Cl_4$) of **3**

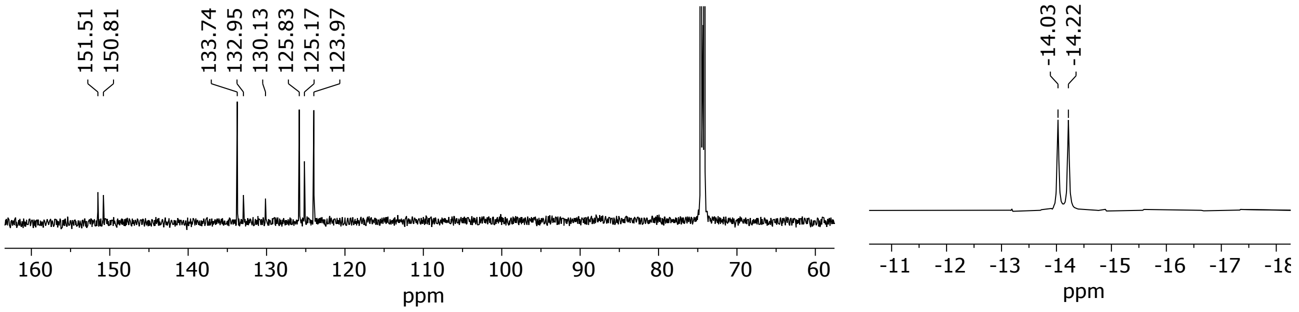

Figure S2.5. ^{13}C-NMR (left) and ^{11}B-NMR (right) spectrum ($C_2D_2Cl_4$) of **3**

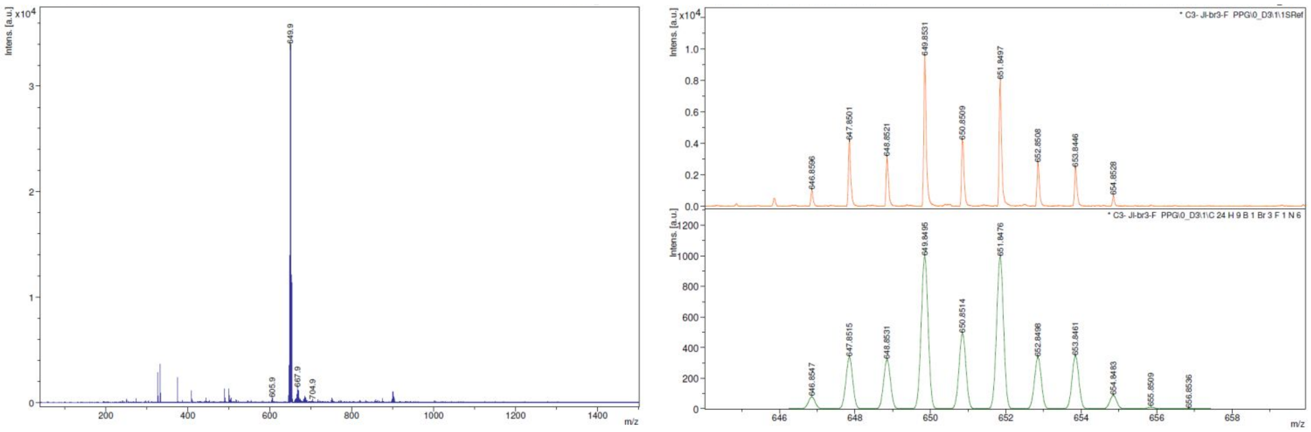

Figure S2.6. MALDI-TOF mass spectrum of **3**.

3. Analysis of intrinsic chirality of SubPcs 1-3

As a consequence of their cone-shaped structure, SubPcs featuring a C_3-symmetric peripheral substitution pattern (as **1**,**2** and **3**) are obtained as a racemic mixture of two enantiomers (*i.e. M* and *P*). In order to assign the *M* or *P* configuration to a C_3-SubPc, the following convention is followed. 1) The enantiopure SubPc is placed having the concave face pointing towards the observer Figure **S3.1.a.**; 2) The carbon atoms bearing the peripheral substituent are identified (denoted as **a**); 3) The carbon atoms that would bear the peripheral substituent in the enantiopure SubPc of opposite chirality are identified (denoted as **b**); 4) If the direction of the path from the carbon atoms **a** to carbon atoms **b** is clockwise, the SubPc is designated as *P*; if the direction of the path is anticlockwise, the SubPc is designated as *M* (Figure **S3.1.b.**).

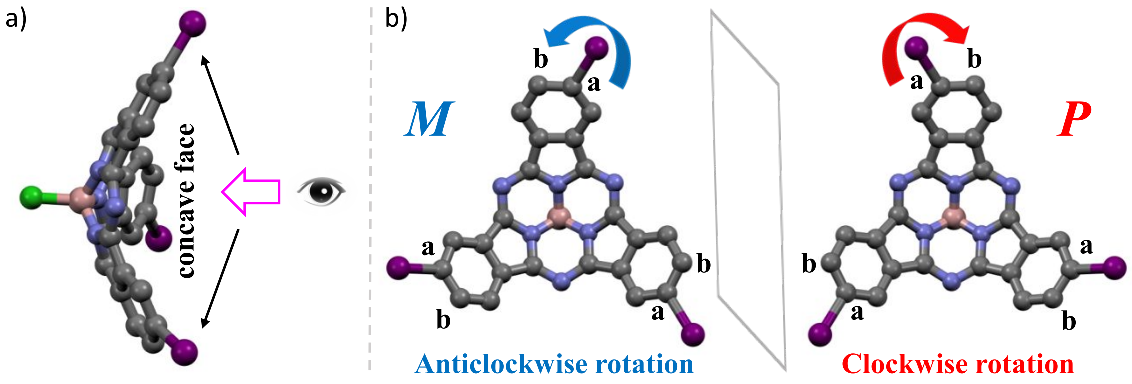

Figure S3.1. *M* and *P* stereodescriptor model for C_3-symmetric chiral SubPcs.

4. Semi-preparative HPLC resolution of racemic SubPcs 1-2 and analysis of 3

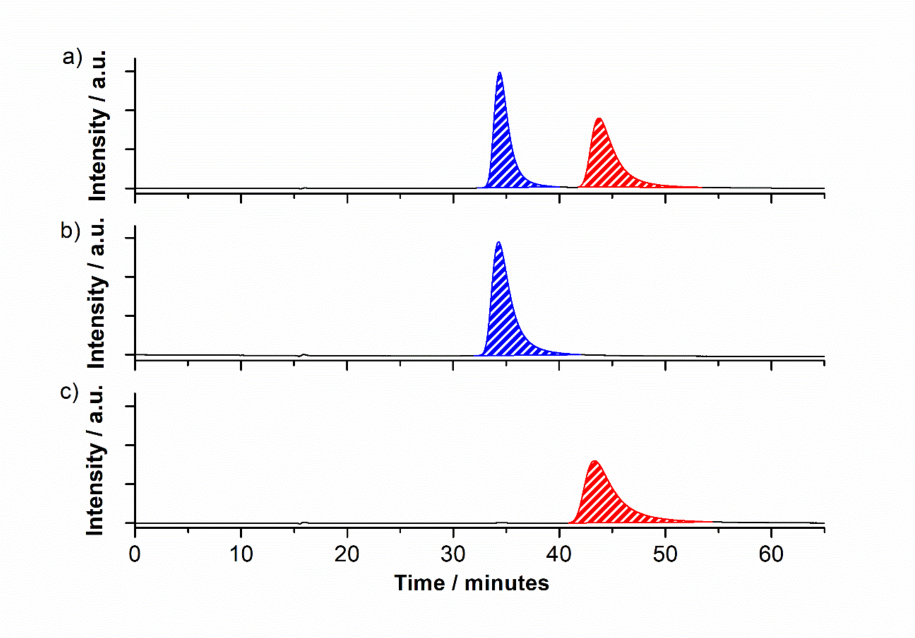

Figure S4.1. a) HPLC chromatogram of a) racemic SubPc **1** with peaks corresponding to *M* enantiomer (blue trace) and *P* enantiomer (red trace). The percentage area underneath the first and second peak is 50.0% and 50.0%, respectively. b) HPLC chromatograms of enantiopure SubPc **1**. c) HPLC chromatograms of enantiopure SubPc **1**. Eluting solvents = toluene/n-hexane 50:50; flow rate = 1.2 mL min−1; temperature = 10 ºC, detection wavelength = 570 nm. The absolute configuration of both the first and the second eluted enantiomers were determined by X-ray crystal structure analysis of the enantiopure SubPcs and corresponds to *M* and *P*, respectively.

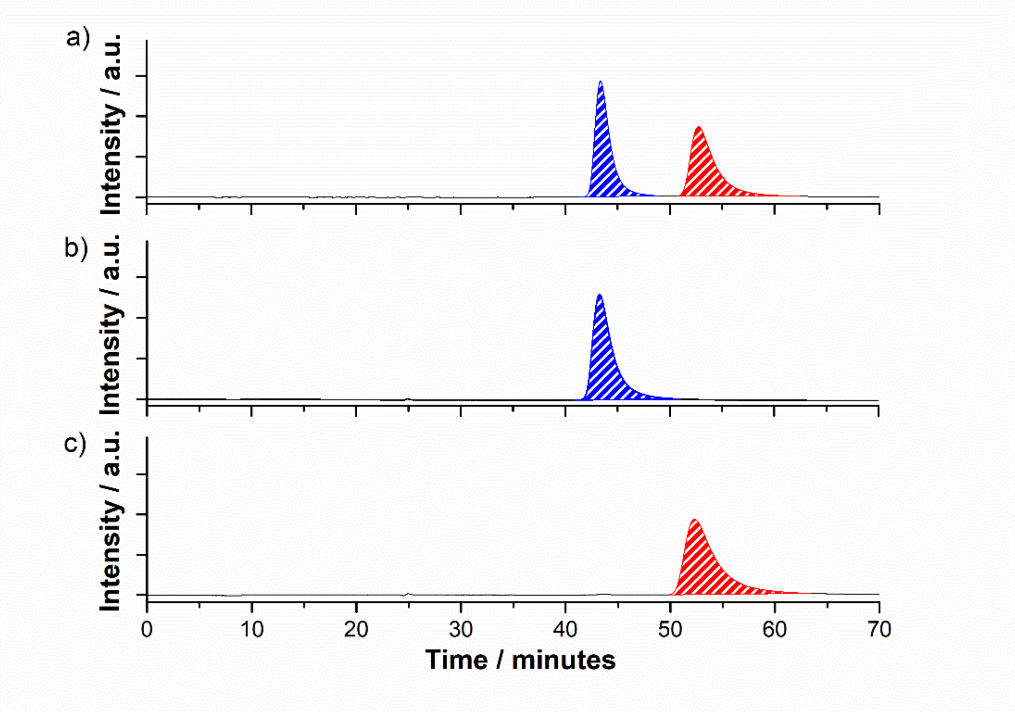

Figure S4.2. a) HPLC chromatogram of a) racemic SubPc **2** with peaks corresponding to *M* enantiomer (blue trace) and *P* enantiomer (red trace). The percentage area underneath the first and second peak is 50.0% and 50.0%, respectively. b) HPLC chromatograms of enantiopure SubPc **2**. c)

HPLC chromatograms of enantiopure SubPc **2**. Eluting solvents = toluene/n-hexane 50:50; flow rate = 1.0 mL min−1; temperature = 10 ºC, detection wavelength = 570 nm. The absolute configuration of both the first and the second eluted enantiomers were determined by X-ray crystal structure analysis of the enantiopure SubPcs and corresponds to *M* and *P*, respectively.

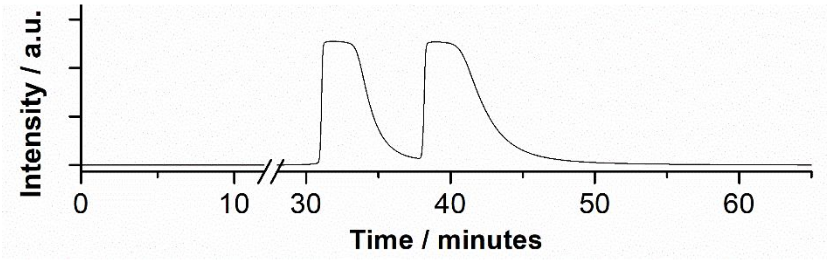

Figure S4.3. HPLC chromatogram of racemic SubPc **1** with peaks corresponding to *M* enantiomer (blue trace) and *P* enantiomer (red trace) in the experimental condition employed for the resolution of racemic SubPc **1** on a semi-preparative scale (concentration of the injected solution: 1.8 mg/mL; injected volume: 200 µL; eluting solvents = toluene/n-hexane 50:50; flow rate = 1.2 mL min^{-1}; temperature = 10 ºC, detection wavelength = 570 nm). Performing runs of 60 minutes, a separation rate of 8.64 mg per day of racemic SubPc **1** can be calculated.

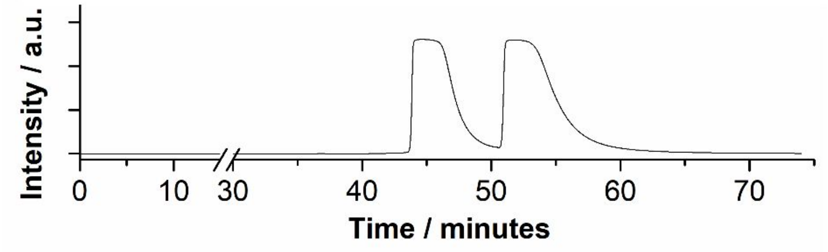

Figure S4.3. HPLC chromatogram of racemic SubPc **2** with peaks corresponding to *M* enantiomer (blue trace) and *P* enantiomer (red trace) in the experimental condition employed for the resolution of racemic SubPc **2** on a semi-preparative scale (concentration of the injected solution: 2.0 mg/mL; injected volume: 200 µL; eluting solvents = toluene/n-hexane 50:50; flow rate = 1.0 mL min^{-1}; temperature = 10 ºC, detection wavelength = 570 nm). Performing runs of 60 minutes, a separation rate of 9.60 mg per day of racemic SubPc **2** can be calculated.

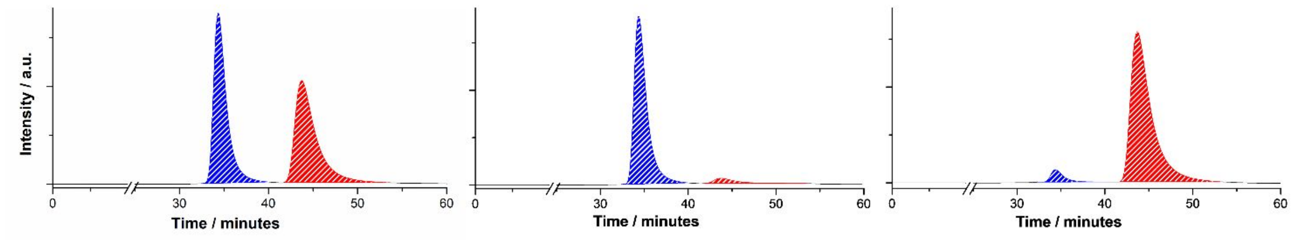

Figure S4.4. a) HPLC chromatogram of a) racemic SubPc **3** with peaks corresponding to *M* enantiomer (blue trace) and *P* enantiomer (red trace). The percentage area underneath the first and second peak is 50.0% and 50.0%, respectively. b) HPLC chromatograms of enantiopure SubPc ***M*3** after axial substitution of ***M*2** with $BF_3.OEt_2$. The percentage area underneath the first and second peak is 94.5 % and 5.5 %, respectively c) HPLC chromatograms of enantiopure SubPc ***P*3** after axial substitution of ***P*2** with $BF_3.OEt_2$. The percentage area underneath the first and second peak is 94.2 % and 5.8 %, respectively. Eluting solvents = toluene/n-hexane 90:10; flow rate = 1.0 mL min−1; temperature = 20 ºC, detection wavelength = 570 nm.

5. UV-vis, circular dichroism and fluorescence spectra of enantiopure SubPcs 1-3

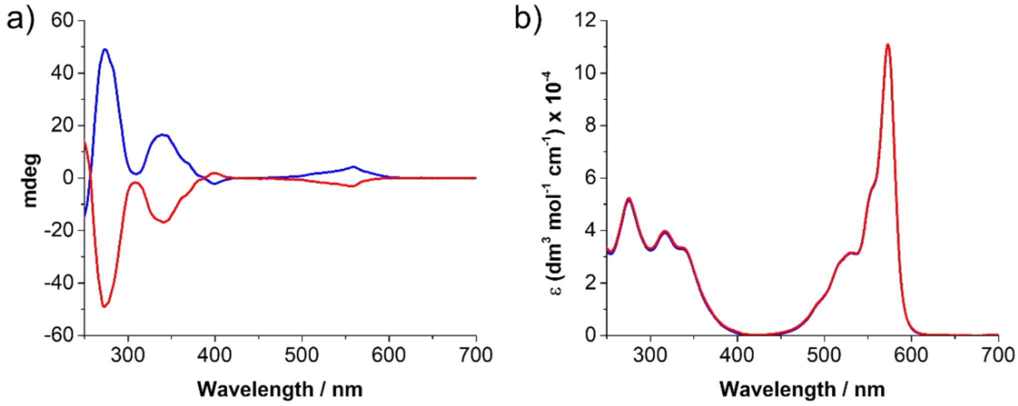

Figure S5.1. a) Circular dichroism spectrum of enantiopure SubPcs ***M*1** (blue spectrum) and ***P*1** (red spectrum) in $CHCl_3$ (concentration = 2 × 10^{-5} M). b) UV-vis absorption spectrum of enantiopure SubPcs ***P*1** (blue spectrum) and ***M*1** (red spectrum) in $CHCl_3$ (concentration = 2 × 10^{-5} M).

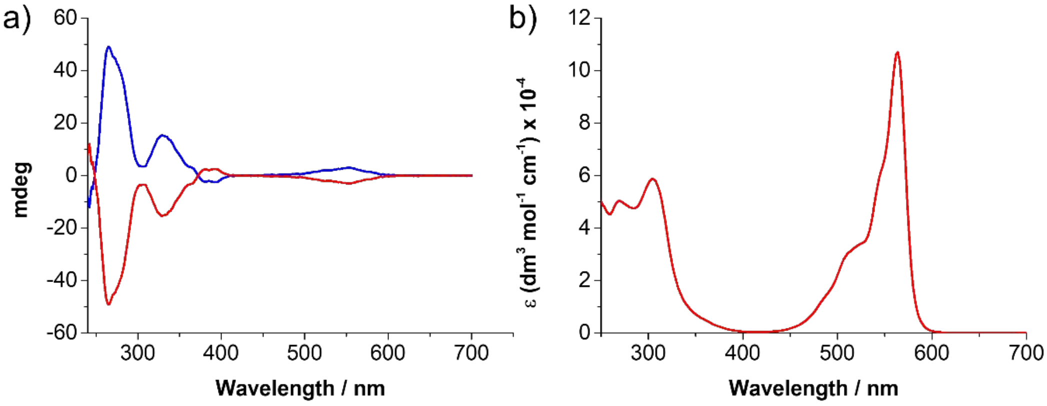

Figure S5.2. a) Circular dichroism spectrum of enantiopure SubPcs ***P*2** (blue spectrum) and ***M*2** (red spectrum) in $CHCl_3$ (concentration = 3.5 × 10^{-5} M). b) UV-vis absorption spectrum of enantiopure SubPcs ***P*1** (blue spectrum) and ***M*1** (red spectrum) in $CHCl_3$ (concentration = 2 × 10^{-5} M).

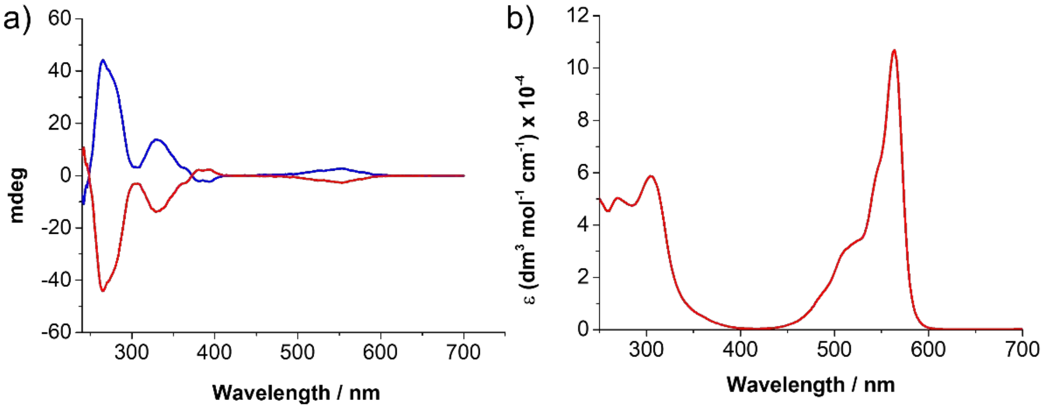

Figure S5.3. a) Circular dichroism spectrum of enantiopure SubPcs ***P*3** (blue spectrum) and ***M*3** (red spectrum) in $CHCl_3$ (concentration = 3.5 × 10^{-5} M). b) UV-vis absorption spectrum of

enantiopure SubPcs ***P*1** (blue spectrum) and ***M*1** (red spectrum) in $CHCl_3$ (concentration = 2×10^{-5} M).

The theoretical CD spectra of the *M* and *P* enantiomers of **1-2** were simulated by TD-DFT using the CAM-B3LYP functional and the standard 6-31G(d) basis set.[3] All structures were previously optimized by DFT at the same level of theory. Analytical harmonic frequencies were computed at the to confirm the nature of the stationary points. All of the calculations were carried out by the methods implemented in Gaussian 16 package.[4]

a)

b)

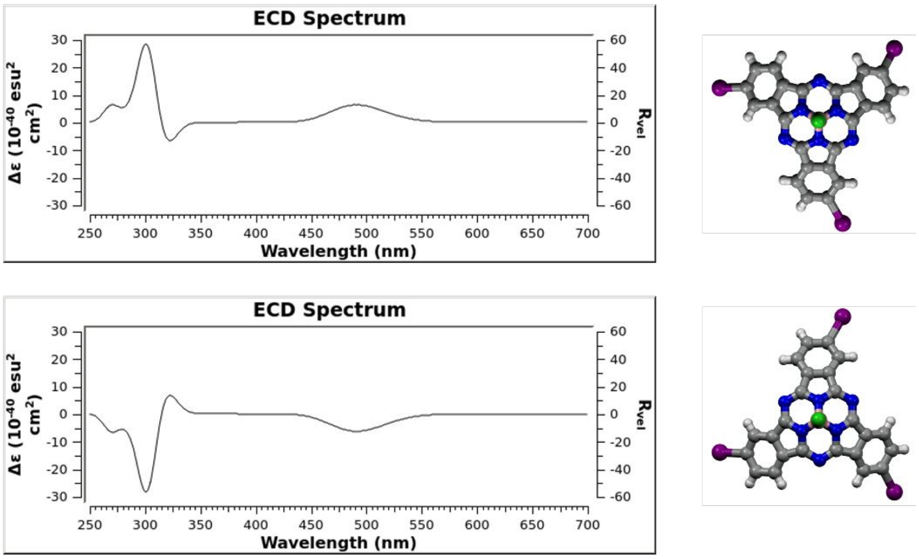

Figure S5.4. Theoretical CD spectra of (a) ***M*1** and (b) ***P*1** simulated by TD-DFT (CAM-B3LYP/6-31G(d)). Similar CD spectra are obtained with ***M*2** and ***P*2**.

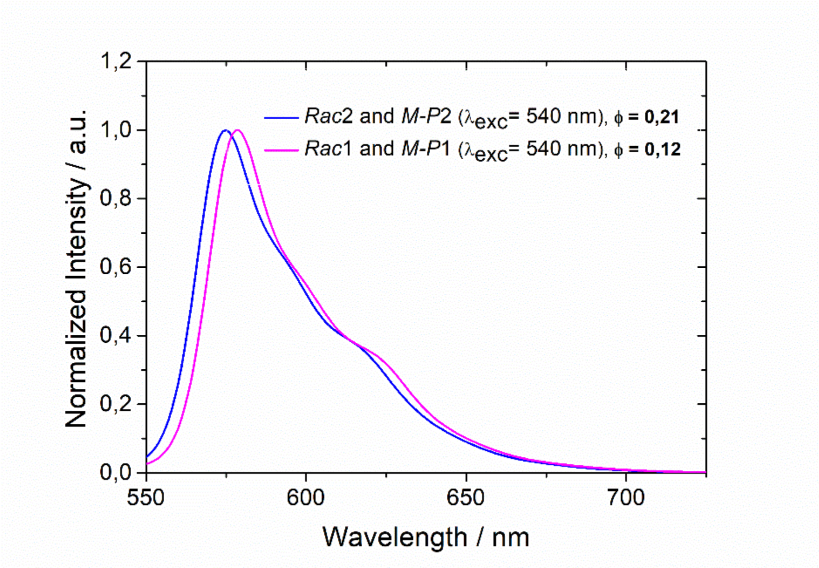

Figure S5.5. Fluorescence spectra of (blue) ***Rac*1** and ***M-P*1** and (pink) ***Rac*2** and ***M-P*2** in $CHCl_3$ (concentration = 3.5×10^{-6} M). Fluorescence quantum yields (ϕ_f) have been calculated using Chloro-dodecafluoroSubPc (Cl-SubPc F_{12}) in benzonitrile ($\phi_f = 0.58$) as reference.

6. X-ray crystal structure of racemic SubPcs 1-2 and enantiopure SubPcs *M*1-2 and *P*1-2

Table 6.1. X-Ray crystallographic data for racemic triiodo-SubPc **1**.

**Chemical formula**	$C_{24}H_9BClI_3N_6O$	
**Formula weight**	824.33 g/mol	
**Temperature**	200(2) K	
**Wavelength**	0.71073 Å	
**Crystal size**	0.020 x 0.020 x 0.085 mm	
**Crystal habit**	dark purple needle	
**Crystal system**	monoclinic	
**Space group**	P 1 21/n 1	
**Unit cell dimensions**	a = 13.6481(5) Å	α = 90°
	b = 7.2351(4) Å	β = 100.829(2)°
	c = 26.4186(13) Å	γ = 90°
**Volume**	2562.3(2) $Å^3$	
**Z**	4	
**Density (calculated)**	2.137 g/cm^3	
**Absorption coefficient**	3.795 mm^{-1}	
**F(000)**	1536	
**Theta range for data collection**	1.57 to 25.35°	
**Index ranges**	-16<=h<=16, -8<=k<=8, -31<=l<=30	
**Reflections collected**	21078	
**Independent reflections**	4681 [R(int) = 0.0619]	
**Coverage of independent reflections**	99.8%	
**Absorption correction**	multi-scan	
**Max. and min. transmission**	0.9280 and 0.7390	
**Refinement method**	Full-matrix least-squares on F^2	
**Refinement program**	SHELXL-2014/7 (Sheldrick, 2014)	
**Function minimized**	Σ w(Fo2 - Fc2)2	
**Data / restraints / parameters**	4681 / 0 / 325	
**Goodness-of-fit on F2**	1.088	
**Δ/σ_{max}**	0.001	
**Final R indices**	3338 data; I>2σ(I)	R1 = 0.0424, wR2 = 0.1046
	all data	R1 = 0.0740, wR2 = 0.1341
**Weighting scheme**	$w=1/[\sigma^2(F_o^2)+(0.0699)^2]$ where $P=(F_o^2+2F_c^2)/3$	
**Largest diff. peak and hole**	1.973 and -1.018 $eÅ^{-3}$	
**R.M.S. deviation from mean**	0.242 $eÅ^{-3}$	

Table S6.2: X-Ray crystallographic data for enantiopure triiodo-SubPc ***M*1**.

**Chemical formula**	$C_{24}H_9BClI_3N_6O$	
**Formula weight**	824.33 g/mol	
**Temperature**	200(2) K	
**Wavelength**	0.71073 Å	
**Crystal size**	0.020 x 0.04 x 0.36 mm	
**Crystal habit**	dark purple needle	
**Crystal system**	monoclinic	
**Space group**	P 1 21/n 1	
**Unit cell dimensions**	a = 13.576(2) Å	α = 90°
	b = 7.2064(11) Å	β = 107.831(7)°
	c = 13.839(2) Å	γ = 90°
**Volume**	1288.9(3) $Å^3$	
**Z**	2	
**Density (calculated)**	2.124 g/cm^3	

**Absorption coefficient**	3.771 mm^{-1}	
**F(000)**	768	
**Theta range for data collection**	1.55 to 25.40°	
**Index ranges**	-15<=h<=16, -7<=k<=8, -16<=l<=16	
**Reflections collected**	8355	
**Independent reflections**	4590 [R(int) = 0.0472]	
**Coverage of independent reflections**	99.6%	
**Absorption correction**	multi-scan	
**Max. and min. transmission**	0.9280 and 0.7390	
**Refinement method**	Full-matrix least-squares on F^2	
**Refinement program**	SHELXL-97 (Sheldrick, 2008)	
**Function minimized**	Σ w(Fo2 - Fc2)2	
**Data / restraints / parameters**	4590 / 1 / 325	
**Goodness-of-fit on F2**	1.016	
**Δ/σ_{max}**	0.001	
**Final R indices**	3254 data; I>2σ(I)	R1 = 0.0437, wR2 = 0.0910
	all data	R1 = 0.0800, wR2 = 0.1295
**Weighting scheme**	$w=1/[\sigma^2(Fo^2)+(0.0593P)^2+0.0000P]$	
**Largest diff. peak and hole**	1.039 and -0.871 $eÅ^{-3}$	
**R.M.S. deviation from mean**	0.210 $eÅ^{-3}$	

Table S6.3: X-Ray crystallographic data for racemic tribromo-SubPc **2**.

**Chemical formula**	$C_{24}H_9BClBr_3N_6O$	
**Formula weight**	786.73 g/mol	
**Temperature**	250(2) K	
**Wavelength**	0.71073 Å	
**Crystal size**	0.024 x 0.042 x 0.116 mm	
**Crystal habit**	purple prismatic	
**Crystal system**	triclinic	
**Space group**	P -1	
**Unit cell dimensions**	a = 8.8637(4) Å	α = 117.2516(16)°
	b = 13.0768(6) Å	β = 96.647(2)°
	c = 13.6479(6) Å	γ = 95.693(2)°
**Volume**	1375.97(11) Å3	
**Z**	2	
**Density (calculated)**	1.899 g/cm3	
**Absorption coefficient**	4.816 mm-1	
**F(000)**	760	
**Theta range for data collection**	1.71 to 25.35°	
**Index ranges**	-10<=h<=10, -15<=k<=15, -16<=l<=16	
**Reflections collected**	25258	
**Independent reflections**	5049 [R(int) = 0.0643]	
**Coverage of independent reflections**	99.9%	
**Absorption correction**	multi-scan	
**Max. and min. transmission**	0.8930 and 0.6050	
**Structure solution technique**	direct methods	
**Structure solution program**	SHELXS-97 (Sheldrick 2008)	
**Refinement method**	Full-matrix least-squares on F2	
**Refinement program**	SHELXL-2014/7 (Sheldrick, 2014)	
**Function minimized**	Σ w(Fo2 - Fc2)2	
**Data / restraints / parameters**	5049 / 0 / 352	
**Goodness-of-fit on F2**	1.031	
**Final R indices**	3376 data; I>2σ(I)	R1 = 0.0459, wR2 = 0.1155
	all data	R1 = 0.0889, wR2 = 0.1504

**Weighting scheme**	w=1/[σ2(Fo2)+(0.0840P)2+0.8741P]<br>where P=(Fo2+2Fc2)/3
**Largest diff. peak and hole**	0.761 and -0.529 eÅ-3
**R.M.S. deviation from mean**	0.123 eÅ-3

Table 6.4.: X-Ray crystallographic data for enantiopure triiodo-SubPc ***M*2**.

**Chemical formula**	$C_{24}H_9BClBr_3N_6O$	
**Formula weight**	667.36 g/mol	
**Temperature**	250(2) K	
**Wavelength**	0.71073 Å	
**Crystal size**	0.034 x 0.056 x 0.122 mm	
**Crystal habit**	purple prismatic	
**Crystal system**	monoclinic	
**Space group**	P 1 21 1	
**Unit cell dimensions**	a = 13.0943(6) Å	α = 90°
	b = 7.2469(3) Å	β = 107.1800(14)°
	c = 13.3072(6) Å	γ = 90°
**Volume**	1206.42(9) Å3	
**Z**	2	
**Density (calculated)**	1.837 g/cm3	
**Absorption coefficient**	5.153 mm-1	
**F(000)**	644	
**Theta range for data collection**	1.60 to 25.35°	
**Index ranges**	-15<=h<=15, -8<=k<=8, -15<=l<=16	
**Reflections collected**	20774	
**Independent reflections**	4409 [R(int) = 0.0476]	
**Coverage of independent reflections**	99.7%	
**Absorption correction**	multi-scan	
**Max. and min. transmission**	0.8440 and 0.5720	
**Structure solution technique**	direct methods	
**Structure solution program**	SHELXS-97 (Sheldrick 2008)	
**Refinement method**	Full-matrix least-squares on F2	
**Refinement program**	SHELXL-2014/7 (Sheldrick, 2014)	
**Function minimized**	Σ w(Fo2 - Fc2)2	
**Data / restraints / parameters**	4409 / 1 / 316	
**Goodness-of-fit on F2**	1.040	
**Final R indices**	3519 data; I>2σ(I)	R1 = 0.0355, wR2 = 0.0822
	all data	R1 = 0.0561, wR2 = 0.1123
**Weighting scheme**	w=1/[σ2(Fo2)+(0.0665P)2]<br>where P=(Fo2+2Fc2)/3	
**Absolute structure parameter**	-0.0(0)	
**Largest diff. peak and hole**	0.469 and -0.432 eÅ-3	
**R.M.S. deviation from mean**	0.152 eÅ-3	

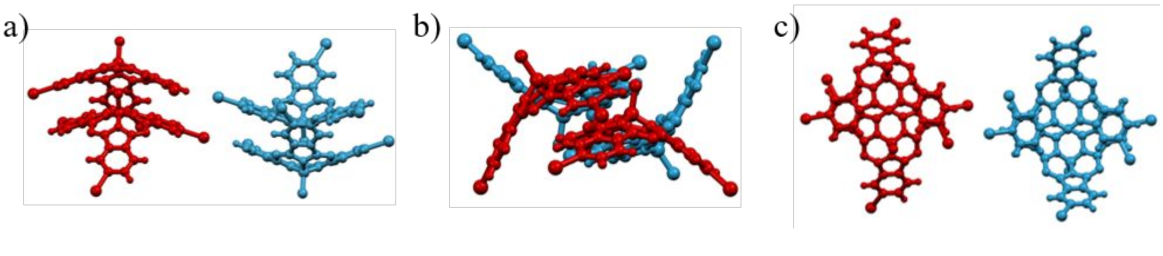

Figure S6.1. Front (a), lateral (b) and top (c) view of a portion of the X-ray crystal structure of racemic SubPc **1** showing the formation of couples of homochiral dimers. In order to facilitate the visualization of the enantiomers, the *P* enantiomer has been colored in red and the *M* enantiomer has been colored in blue. Water molecules of crystallization and hydrogen atoms have been omitted for clarity.

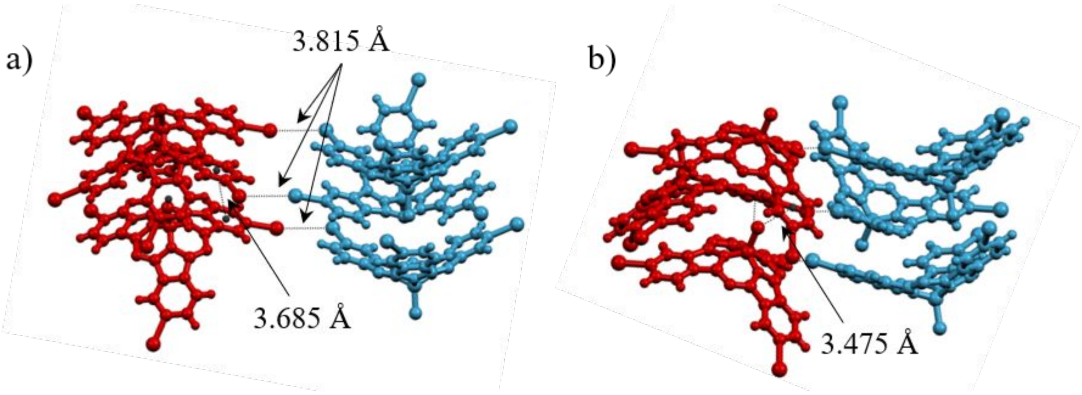

Figure S6.2. Two different views of a portion of the X-ray crystal structure of racemic SubPc **1** evidencing the interactions within the homochiral columns and between adjacent homochiral columns composed of SubPcs of opposite chirality. In a) the distance between the two centroids of the six-membered aromatic rings of one of the isoindole units of vicinal SubPcs in the same column (3.685 Å) and the distances between couples of iodine atoms of different SubPcs in adjacent columns (3.815 Å) has been marked with a dashed black line. In b) the distance between the apical chlorine ligand and the centroid of the six-membered aromatic ring of one of the isoindole units of SubPcs belonging to different stacked dimers (3.475 Å) has been marked with a dashed black line. In order to facilitate the visualization of the enantiomers, the *P* enantiomer has been colored in red and the *M* enantiomer has been colored in blue. Water molecules of crystallization and hydrogen atoms have been omitted for clarity.

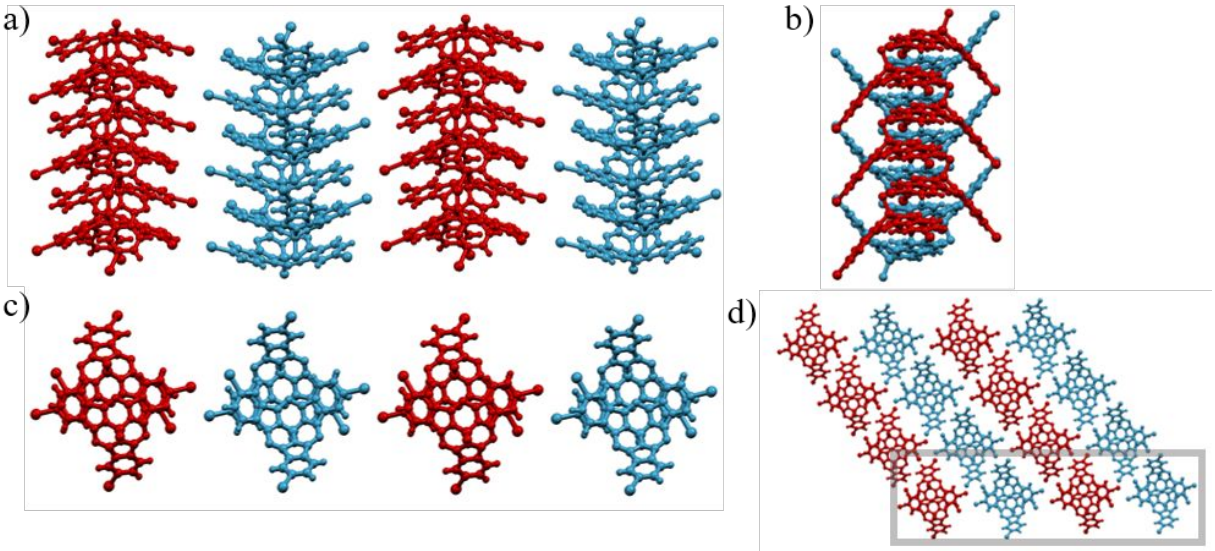

Figure S6.3. Front (a), lateral (b) and top (c) view of the portion of the X-ray crystal structure of racemic SubPc **1** framed in d) by a grey border showing the antiparallel arrangement adopted by alternating homochiral columns composed of molecules of opposite chirality. In order to facilitate the visualization of the enantiomers, the *P* enantiomer has been colored in red and the M

enantiomer has been colored in blue. Water molecules of crystallization and hydrogen atoms have been omitted for clarity.

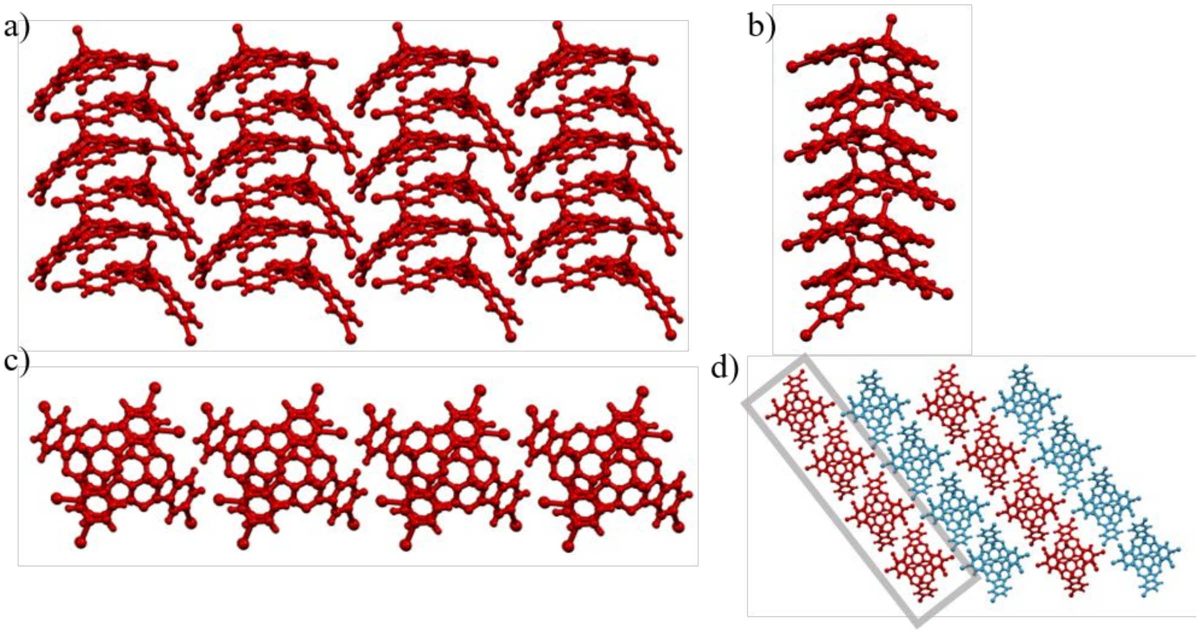

Figure S6.4. Front (a), lateral (b) and top (c) view of the portion of the X-ray crystal structure of racemic SubPc **1** framed in d) by a grey border showing the parallel arrangement adopted by adjacent homochiral columns composed of molecules of the same chirality. In order to facilitate the visualization of the enantiomers, the *P* enantiomer has been colored in red and the M enantiomer has been colored in blue. Water molecules of crystallization and hydrogen atoms have been omitted for clarity.

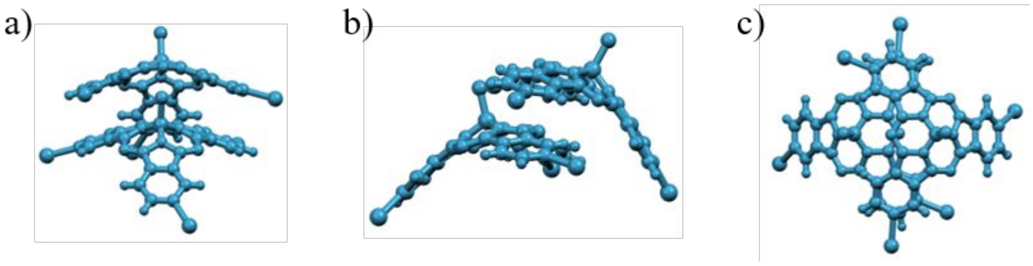

Figure S6.5. Front (a), lateral (b) and top (c) view of a portion of the X-ray crystal structure of enantiopure SubPc ***M*1** showing the formation of homochiral dimers. Water molecules of crystallization and hydrogen atoms have been omitted for clarity.

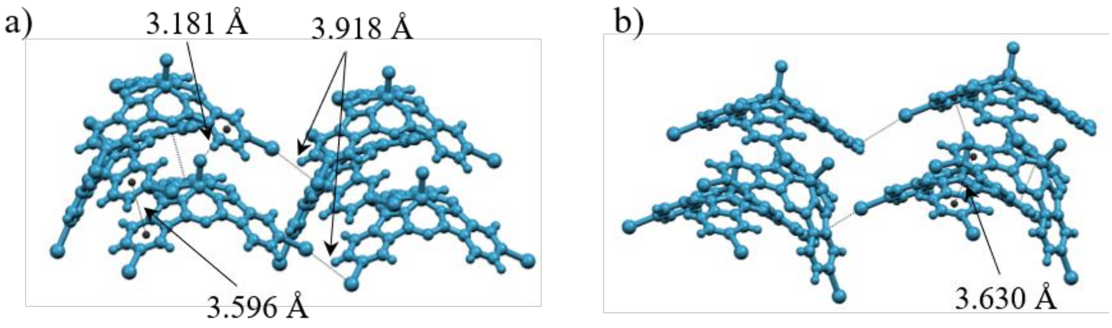

Figure S6.6. Two different views of a portion of the X-ray crystal structure of enantiopure SubPc ***M*1** evidencing the interactions within and between vicinal columns. In a) the distance between the two centroids of the six-membered aromatic rings of one of the isoindole units of vicinal SubPcs in the same column (3.596 Å), the distance between the apical chlorine ligand and the centroid of the six-membered aromatic ring of one of the isoindole units of SubPsc belonging to the adjacent stacked dimers (3.181 Å) and the distances between couples of iodine atoms of different SubPcs in adjacent columns (3.918 Å) has been marked with a dashed black line. In b) the distance between the two centroids of the six-membered aromatic rings of one of the isoindole units of vicinal SubPcs in the same column (3.630 Å) has been marked with a dashed black line. Water molecules of crystallization and hydrogen atoms have been omitted for clarity.

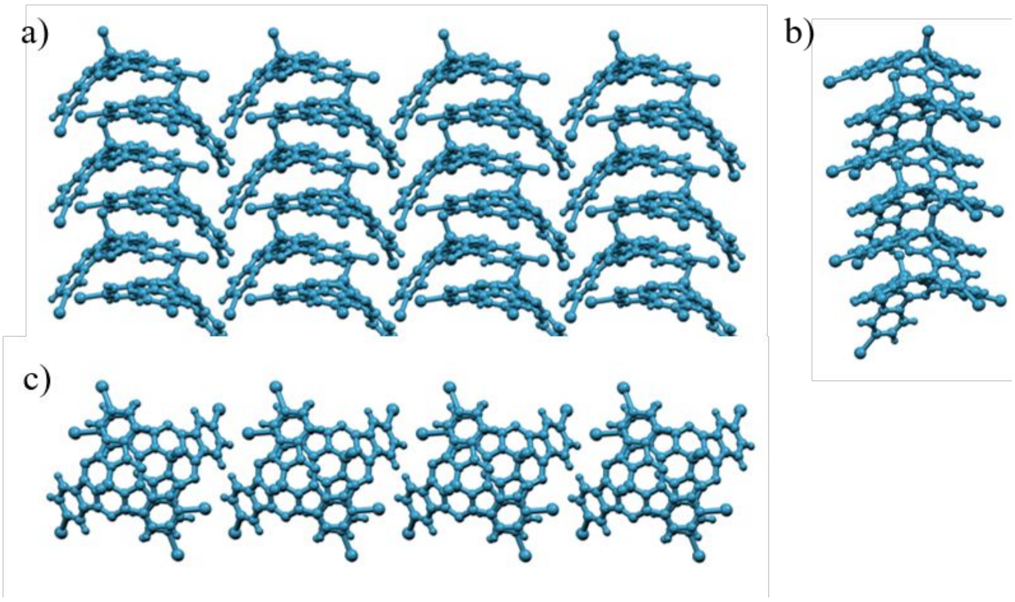

Figure S6.7. Front (a), lateral (b) and top (c) view of the X-ray crystal packing of SubPc ***M*1**. In order to facilitate the visualization of the enantiomers, the *P* enantiomer has been colored in red and the *M* enantiomer has been colored in blue. Water molecules of crystallization and hydrogen atoms have been omitted for clarity.

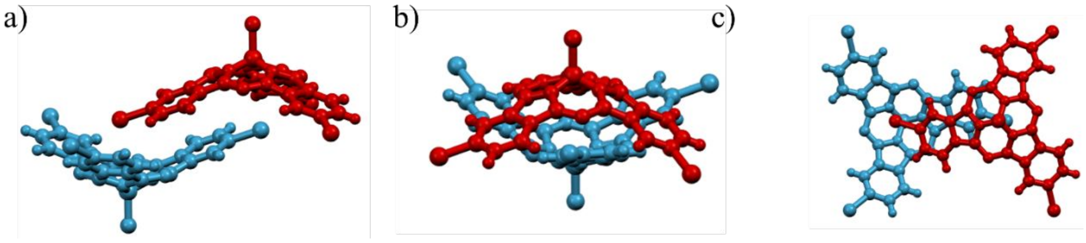

Figure S6.8. Front (a), lateral (b) and top (c) view of a portion of the X-ray crystal structure of racemic SubPc **2** showing the formation of couples of heterochiral dimers. In order to facilitate the visualization of the enantiomers, the *P* enantiomer has been colored in red and the *M* enantiomer has been colored in blue. Solvent molecules of crystallization and hydrogen atoms have been omitted for clarity.

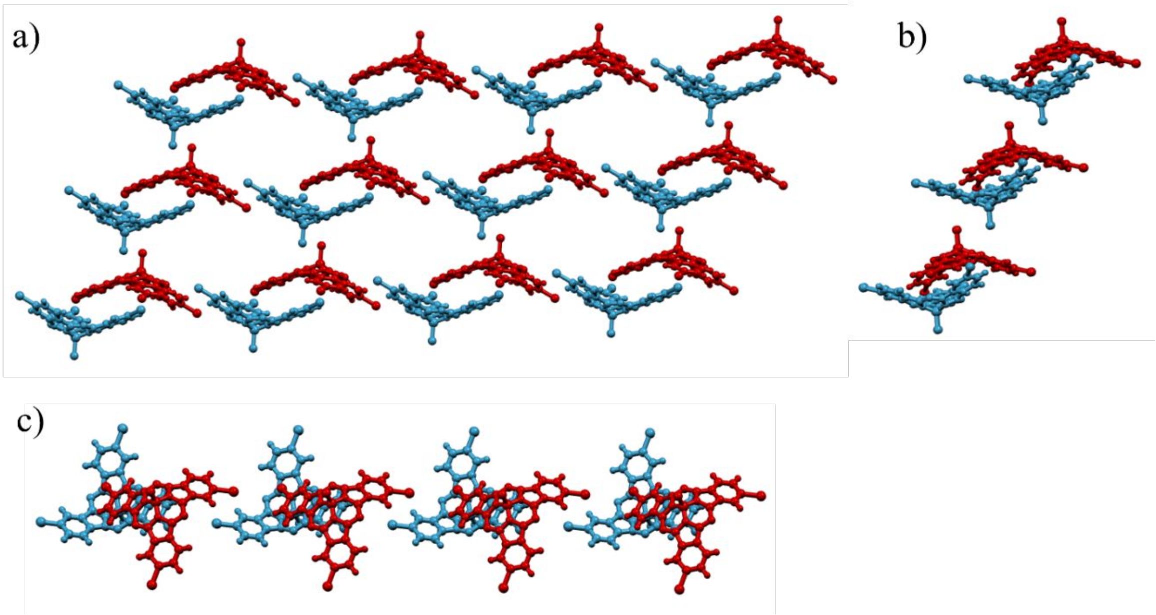

Figure S6.9. Front (a), lateral (b) and top (c) view of the X-ray crystal packing of racemic SubPc **2**. In order to facilitate the visualization of the enantiomers, the *P* enantiomer has been colored in red and the M enantiomer has been colored in blue. Solvent molecules of crystallization and hydrogen atoms have been omitted for clarity.

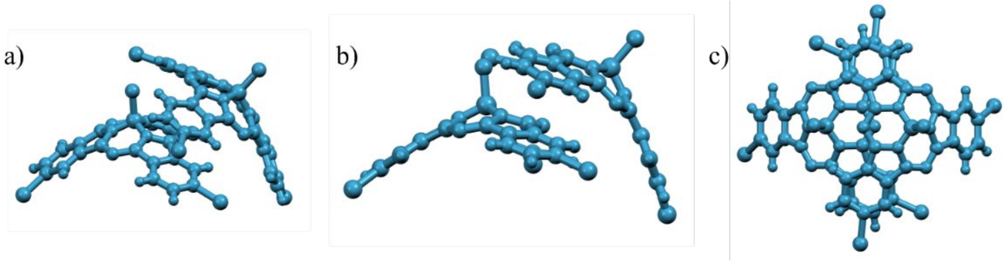

Figure S6.10. Front (a), lateral (b) and top (c) view of a portion of the X-ray crystal structure of enantiopure SubPc ***M*2** showing the formation of homochiral dimers. Water molecules of crystallization and hydrogen atoms have been omitted for clarity.

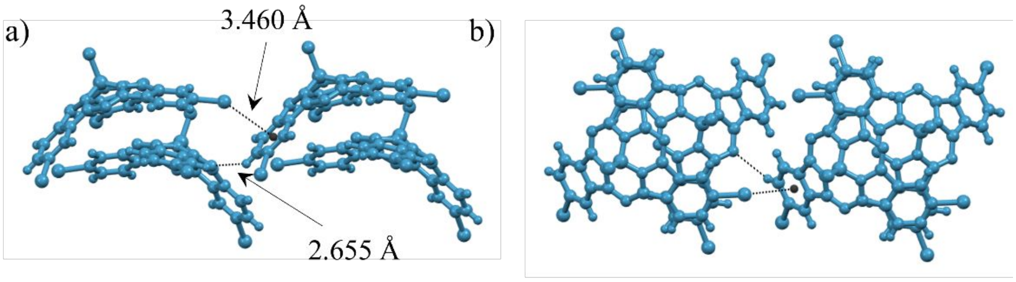

Figure S6.11. Two different views of a portion of the X-ray crystal structure of enantiopure SubPc ***M*2** evidencing the interactions within and between vicinal columns. In a) the distance between the two centroids of the six-membered aromatic rings of one of the isoindole units of vicinal SubPcs in the same column (3.596 Å), the distance between the apical chlorine ligand and the centroid of the six-membered aromatic ring of one of the isoindole units of SubPsc belonging to the adjacent stacked dimers (3.181 Å) and the distances between couples of iodine atoms of different SubPcs in adjacent columns (3.918 Å) has been marked with a dashed black line. In b) the distance between

the two centroids of the six-membered aromatic rings of one of the isoindole units of vicinal SubPcs in the same column (3.630 Å) has been marked with a dashed black line. Water molecules of crystallization and hydrogen atoms have been omitted for clarity.

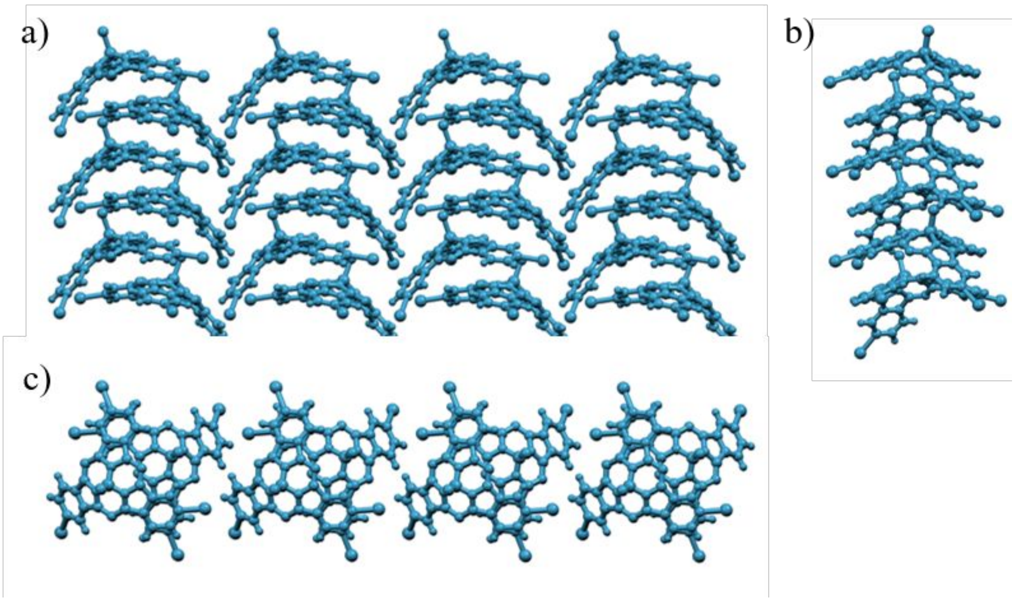

Figure S6.12. Front (a), lateral (b) and top (c) view of the X-ray crystal packing of SubPc ***M*2**. In order to facilitate the visualization of the enantiomers, the *P* enantiomer has been colored in red and the M enantiomer has been colored in blue. Water molecules of crystallization and hydrogen atoms have been omitted for clarity.

7. On-surface organization and polymerization of racemic SubPcs 1-3 and enantiopure SubPcs *M*1-3 and *P*1-3

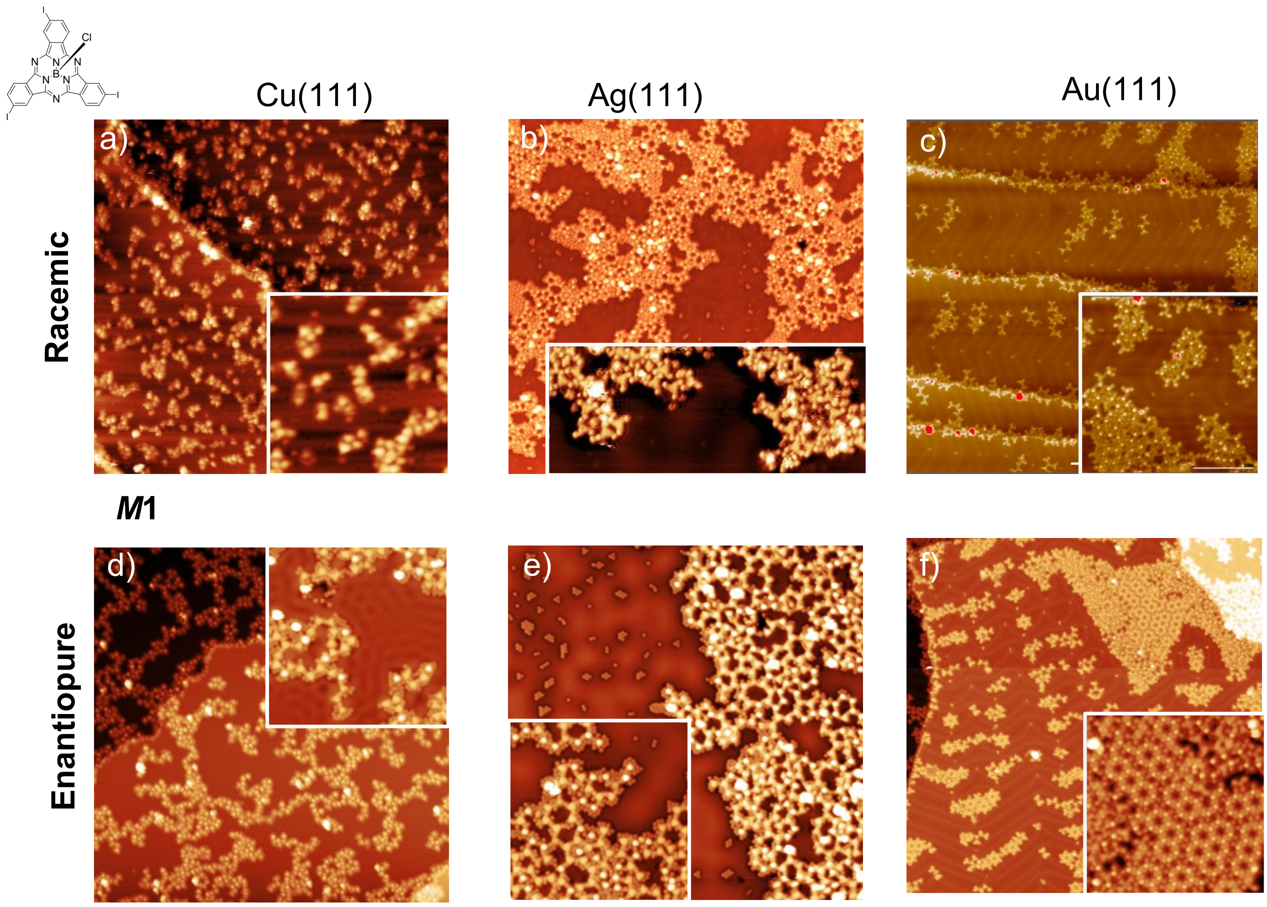

Figure S7.1. STM images of (a-c) ***Rac*1** and (d-e) ***M*1** on the three noble metal surfaces deposited at RT. Only in the case of Au(111) we find some ordering because, contrarily to Cu(111) and Ag(111), most of the molecules maintain their integrity after adsorption. Such order is visibly better for the enantiopure case (f). STM image details: a) V_{bias} = 1.0 V, I_s = 50 pA, size = 100x100 nm^2 (inset: V_{bias} = -0.5 V, I_s = 25 pA, size = 25x25 nm^2); b) V_{bias} = -1.0 V, I_s = 100 pA, size = 100x100 nm^2 (inset: V_{bias} = -0.3 V, I_s = 50pA, size = 25x10 nm^2); c) V_{bias} = -1.0 V, I_s = 70 pA, size = 100x100 nm^2 (inset: V_{bias} = -0.5 V, I_s = 70 pA, size = 30x30 nm^2); d) V_{bias} = 1.0 V, I_s = 100 pA, size = 100x100 nm^2 (inset: V_{bias} = -0.1 V, I_s = 100 pA, size = 20x20 nm^2); e) V_{bias} = -0.1 V, I_s = 100 pA, size = 50x50 nm^2 (inset: V_{bias} = -0.1 V, I_s = 100 pA, size = 20x20 nm^2); f) V_{bias} = -1.0 V, I_s = 70 pA, size = 100x100 nm^2 (inset: V_{bias} = 0.1 V, I_s = 100 pA, size = 20x20 nm^2).

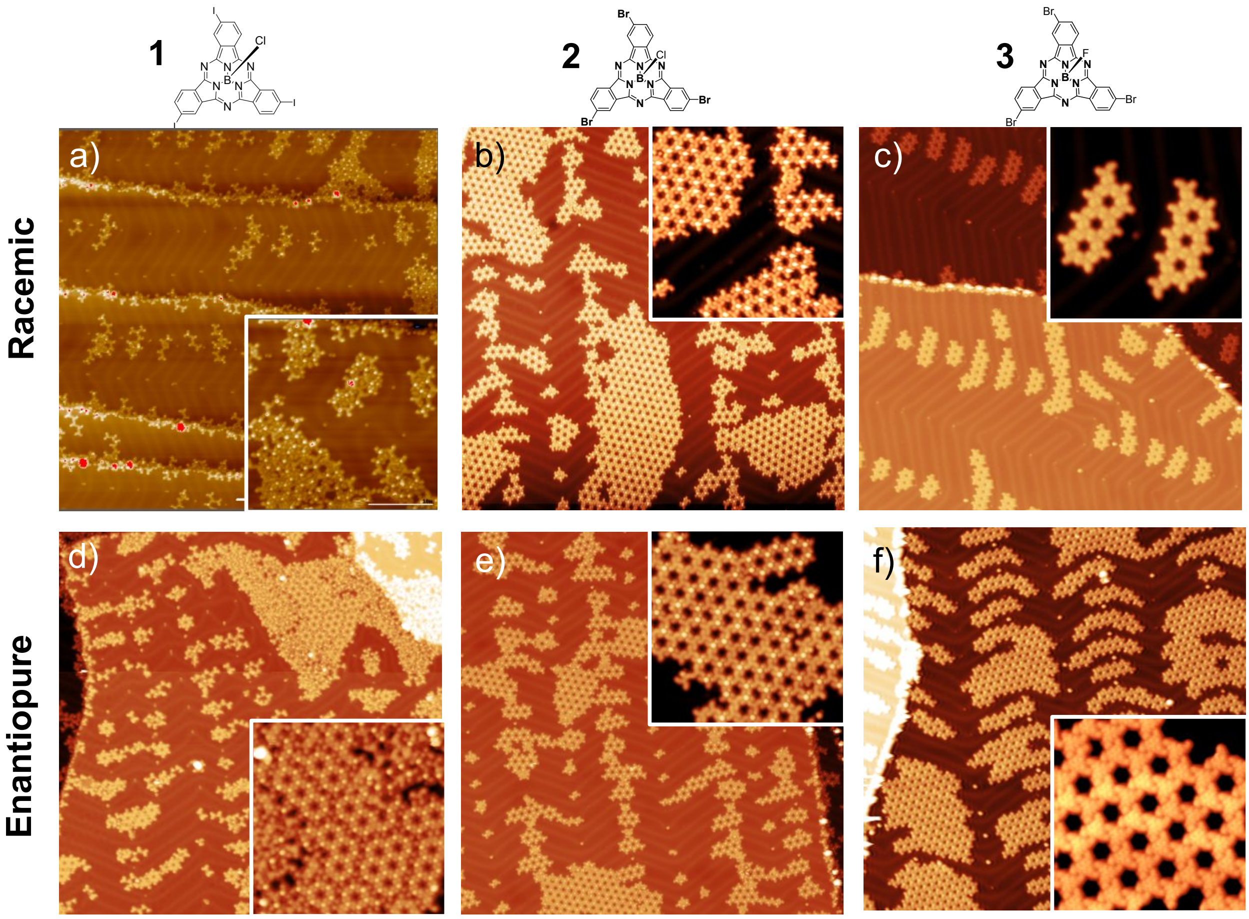

Figure S7.2. STM images of (a-c) ***Rac*1- 3** and ***M*1-3** (d-f) after deposition at RT on Au(111). The structures are more regular using external Br, since these halogens do not split during deposition. Note that the regular porous structures are chiral selective for the three molecules. Moreover, in the case of enantiopure ***P*1-3** compounds we obtain identical results to the ones shown in the bottom row**.** STM image details: a) V_{bias} = -1.0 V, I_s = 70 pA, size = 100x100 nm^2 (inset: V_{bias} = -0.5 V, I_s = 70 pA, size = 30x30 nm^2); b) V_{bias} = -1.0 V, I_s = 60 pA, size = 100x100 nm^2 (inset: V_{bias} = -1.0 V, I_s = 60 pA, size = 25x25 nm^2); c) V_{bias} = -1.0 V, I_s = 50 pA, size = 100x100 nm^2 (inset: V_{bias} = -1.5 V, I_s = 100 pA, size = 15x15 nm^2); d) V_{bias} = -1.0 V, I_s = 70 pA, size = 100x100 nm^2 (inset: V_{bias} = 0.1 V, I_s = 100 pA, size = 20x20 nm^2); e) V_{bias} = -1.0 V, I_s = 100 pA, size = 100x100 nm^2 (inset: V_{bias} = -1.0 V, I_s = 100 pA, size = 20x20 nm^2); f) V_{bias} = -1.0 V, I_s = 100 pA, size = 100x100 nm^2 (inset: V_{bias} = -1.5 V, I_s =200 pA, size = 10x10 nm^2).

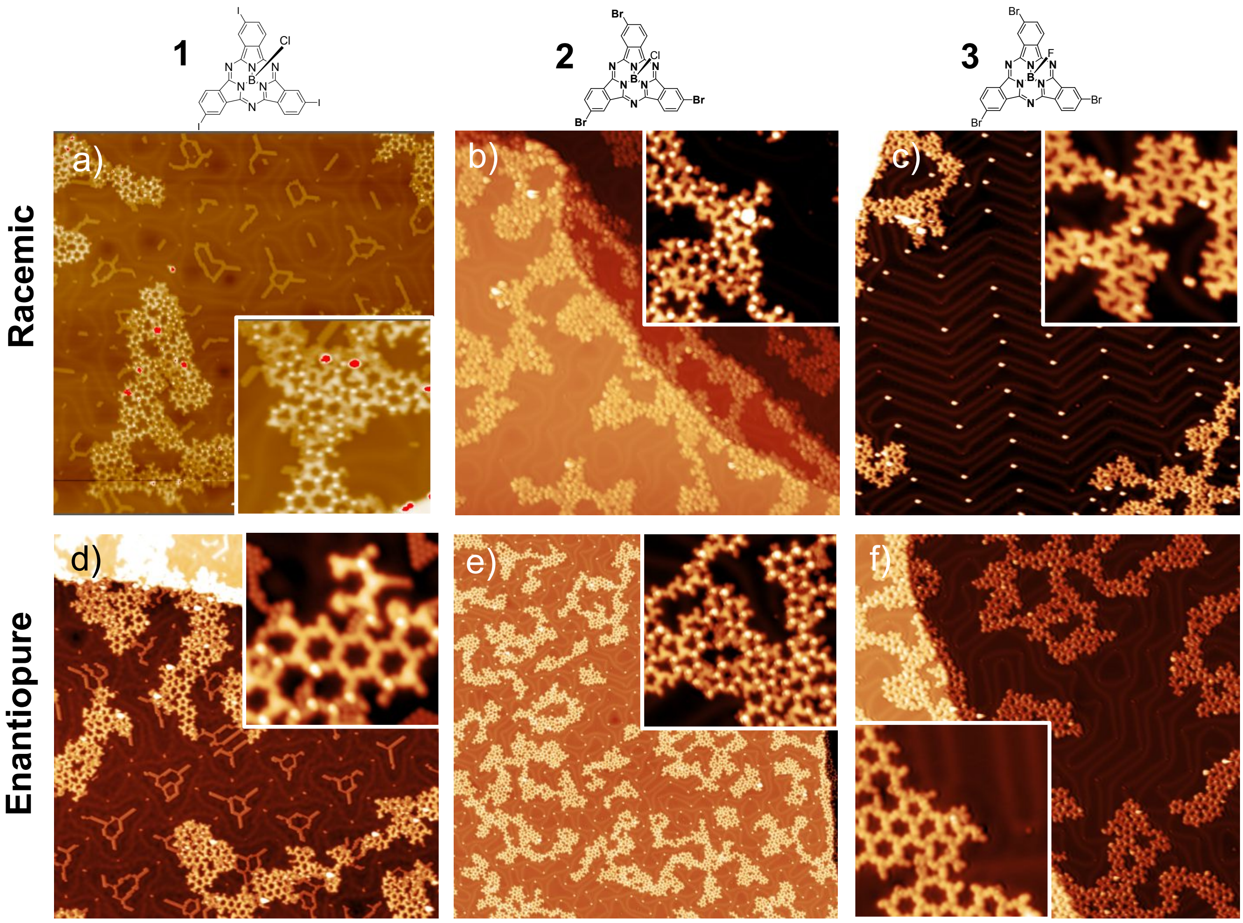

Figure S7.3. STM images of ***Rac*1-3** (a-c) and ***M*1-3** (d-e) imaged at 4K after annealing above 200ºC on Au(111). Annealing after room temperature deposition leads to irregular structures probably due to hindering effects by the split external halogen atoms. Note that these halogen atoms are found as dimer spheres on the surface, generally disrupting the "herringbone" resconstruction of the pristine Au(111) surface. Despite this, the order is much better for the enantiopure cases. STM image details: a) V_{bias} = -0.5 V, I_s = 100 pA, size = 100x100 nm^2 (inset: V_{bias} = -0.5V, I_s = 70 pA, size = 22x22 nm^2); b) V_{bias} = -1.0 V, I_s = 100 pA, size = 100x100 nm^2 (inset: V_{bias} = -1.0 V, I_s = 50 pA, size = 20x20 nm^2); c) V_{bias} = -1.0 V, I_s = 100 pA, size = 100x100 nm^2 (inset: V_{bias} = -1.0 V, I_s = 100 pA, size = 20x20 nm^2); d) V_{bias} = -0.1 V, I_s = 100 pA, size = 100x100 nm^2 (inset: V_{bias} = -0.1 V, I_s = 100 pA, size = 11x11 nm^2); e) V_{bias} = -1.0 V, I_s = 100 pA, size = 200x200 nm^2 (inset: V_{bias} = -1.0 V, I_s = 100 pA, size = 20x20 nm^2); f) V_{bias} = -1.0 V, I_s = 100 pA, size = 100x100 nm^2 (inset: V_{bias} = 0.2 V, I_s = 100 pA, size = 15x15 nm^2).

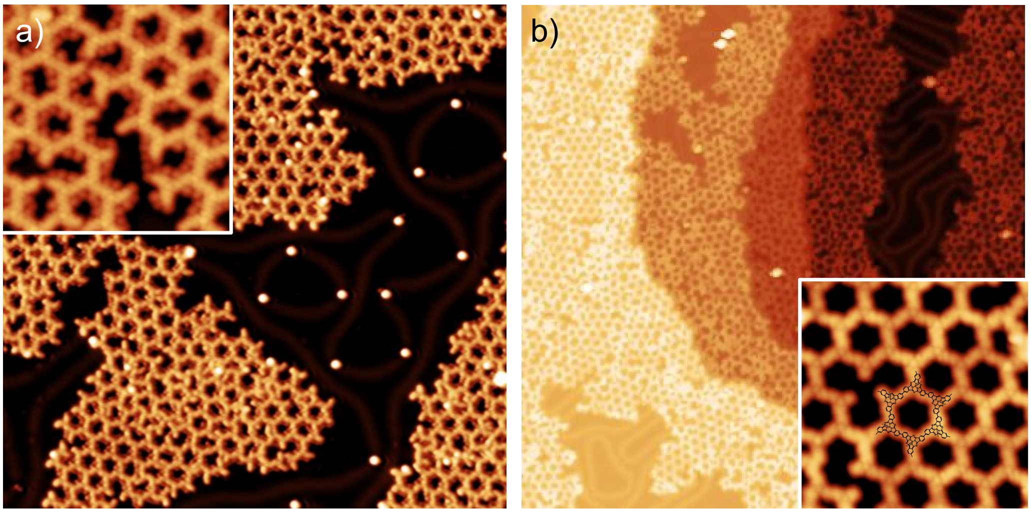

Figure S7.4. Another polymerization attempt of ***M*3** similar to Fig. 5b-c of the main manuscript imaged at 4K after high temperature deposition on Au(111). a) Overview and zoom after 90 min. deposition with substrate kept at 135ºC. The cleaved Br atoms are identified around the polymeric structures. b) Post annealing above 300ºC resulting in activation of non-reacted species and desorption of the Br atoms from the surface. The final order is similar to Fig. 5c, indicating that high temperature deposition at low molecular fluxes is key for improved 2D polymerization by on-surface synthesis. STM image details: a) V_{bias} = -1.0 V, I_s = 90 pA, size = 50x50 nm^2 (inset: V_{bias} = -1.0V, I_s = 120 pA, size = 10x10 nm^2); b) V_{bias} = -1.0 V, I_s = 90 pA, size = 100x100 nm^2 (inset: V_{bias} = -1.0 V, I_s = 90 pA, size = 10x10 nm^2).

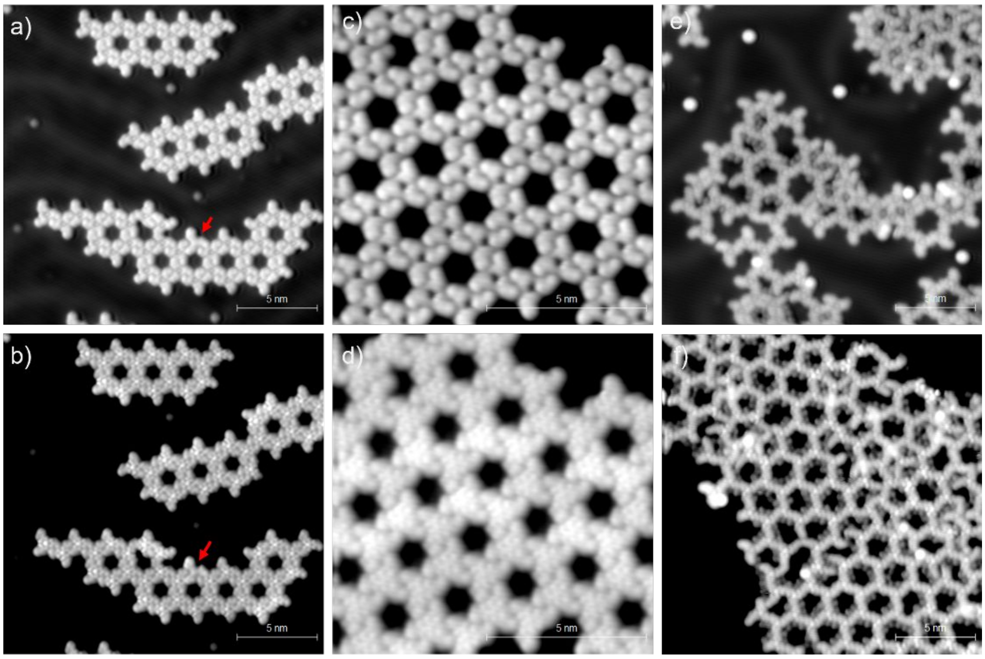

Figure S7.4. a) and b) High-resolution image of ***P*3** deposited at RT on Au(111) imaged with two different setpoints. The axial ligand is present on all molecules, except on the one marked with the red arrow. c) and d) High resolution image of ***M*3** deposited at RT on Au(111) imaged with two different setpoints. Within this island, we observe the three-fold symmetry in the molecules, evidencing identical size of the propeller legs. Note that depending on the tip termination the B-F axial ligand can be concealed, even when using similar experimental conditions as panels a and b.

e) and f) ***M*3** polymerization on Au(111) achieved using the low deposition rate (over 90 min.) with the substrate kept at 135°C. The axial ligand (B-F) is visible in practically all the hexagonal corners, demonstrating its recurrent presence on the molecules. Moreover, this axial ligand imaging suggests bowl-down predominance of the molecular species when forming the covalent bonds of the polymers. The faint spheres within the pores correspond to Br atoms attaching next to the polymeric structures. STM details: a) 100mV, 250pA; b) -1.50V, 250pA; c) 100mV, 200pA; d) -1.5V, 200pA; e) -1.0 V, 120pA; f) -1.0 V, 120pA.

8. Theoretical calculations.

Theoretical calculations were performed with the Density Functional Theory (DFT), using the OPTPBE functional[5] and imposing periodic boundary conditions. The electronic density was expanded in a plane-wave basis and the interaction between electrons and nuclei was described with the projector-augmented wave (PAW) pseudopotentials from the VASP database. We have used a cutoff energy of 420 eV for the plane wave basis and the electron density was optimized until a convergence of 10^{-6} eV. For geometry optimizations, we imposed all the Hellmann-Feynman forces to be lower than 0.01 eV/Å. The Au(111) surface was modeled as a supercell 4 layers of 7x7 Au atoms (196 Au atoms) and including a vacuum of ~23 Å, in order to avoid spurious interactions with the closest replica. Due to the size of the system, reciprocal space was sampled with the Γ-point. All calculations were carried out using the Vienna Ab Initio Simulation Package (VASP).[6]

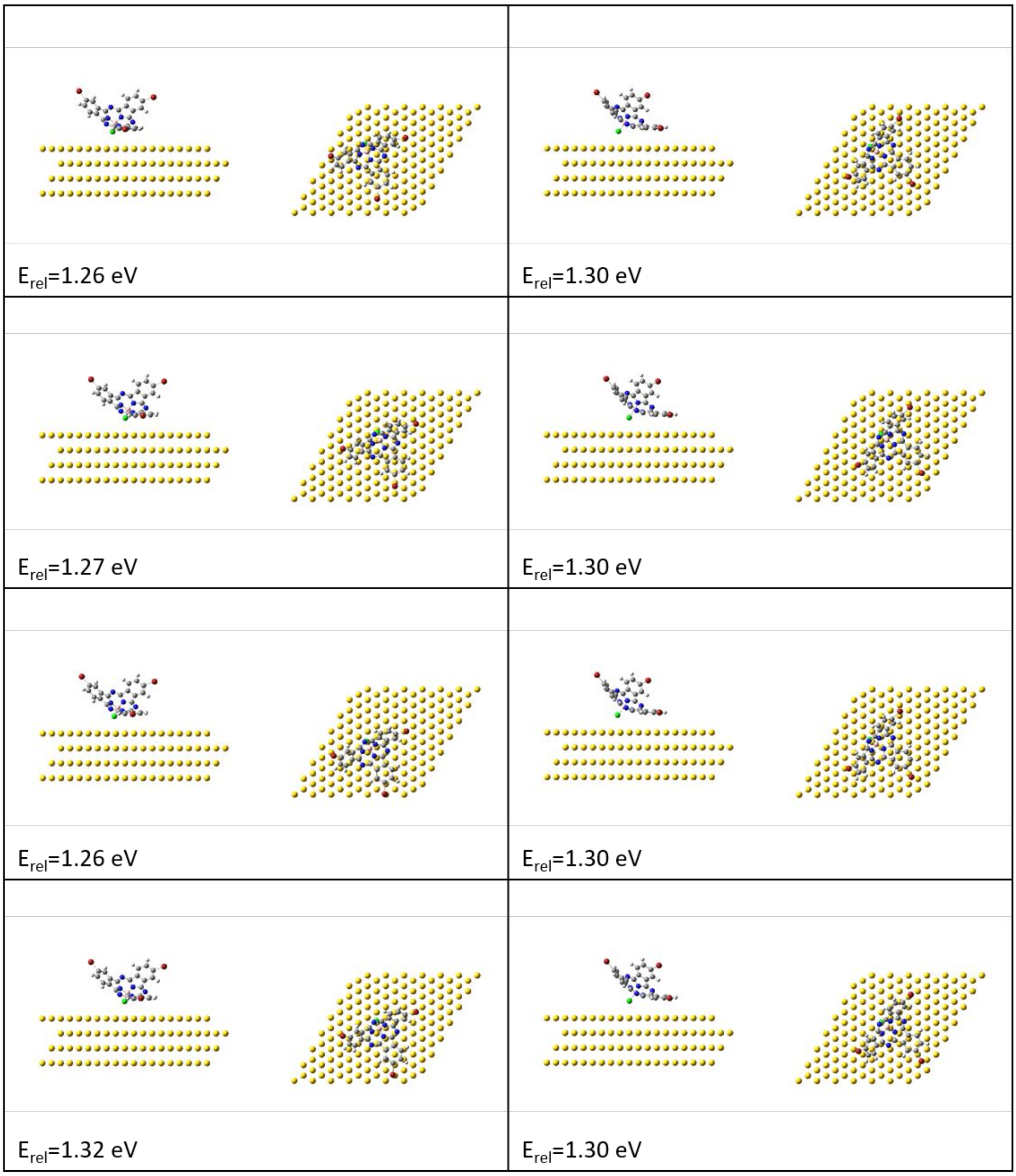

Figure S8.1. Side and top view of all computed structures in the adsorption of ***M*2** on the Au(111) surface. Several structures have been considered for each configuration (*Cl-down, Cl-up, Cl-tiltedA, Cl-tiltedB, DeCl-down* and *DeCl-up*), see main text for details.

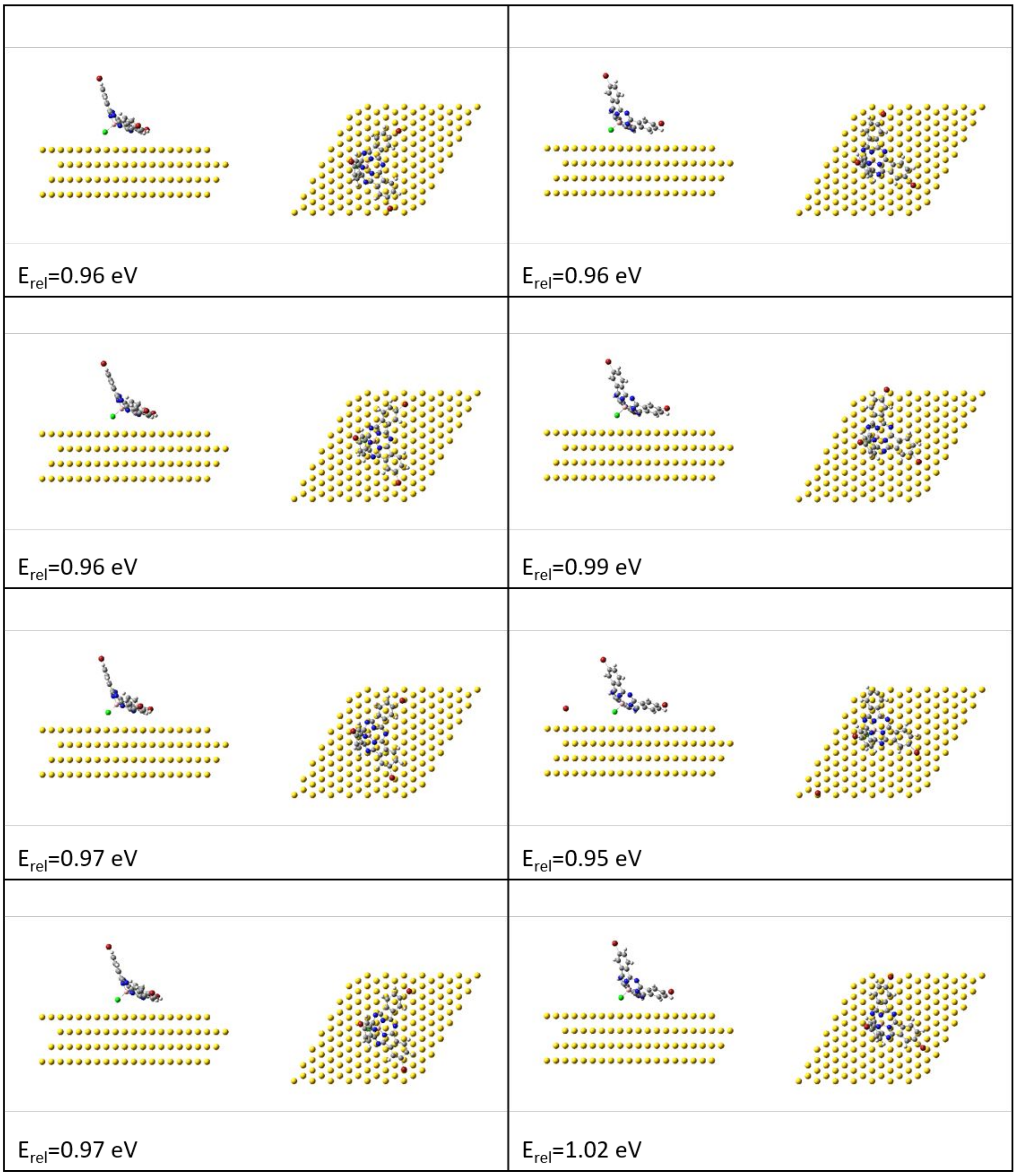

Figure S8.1-cont.

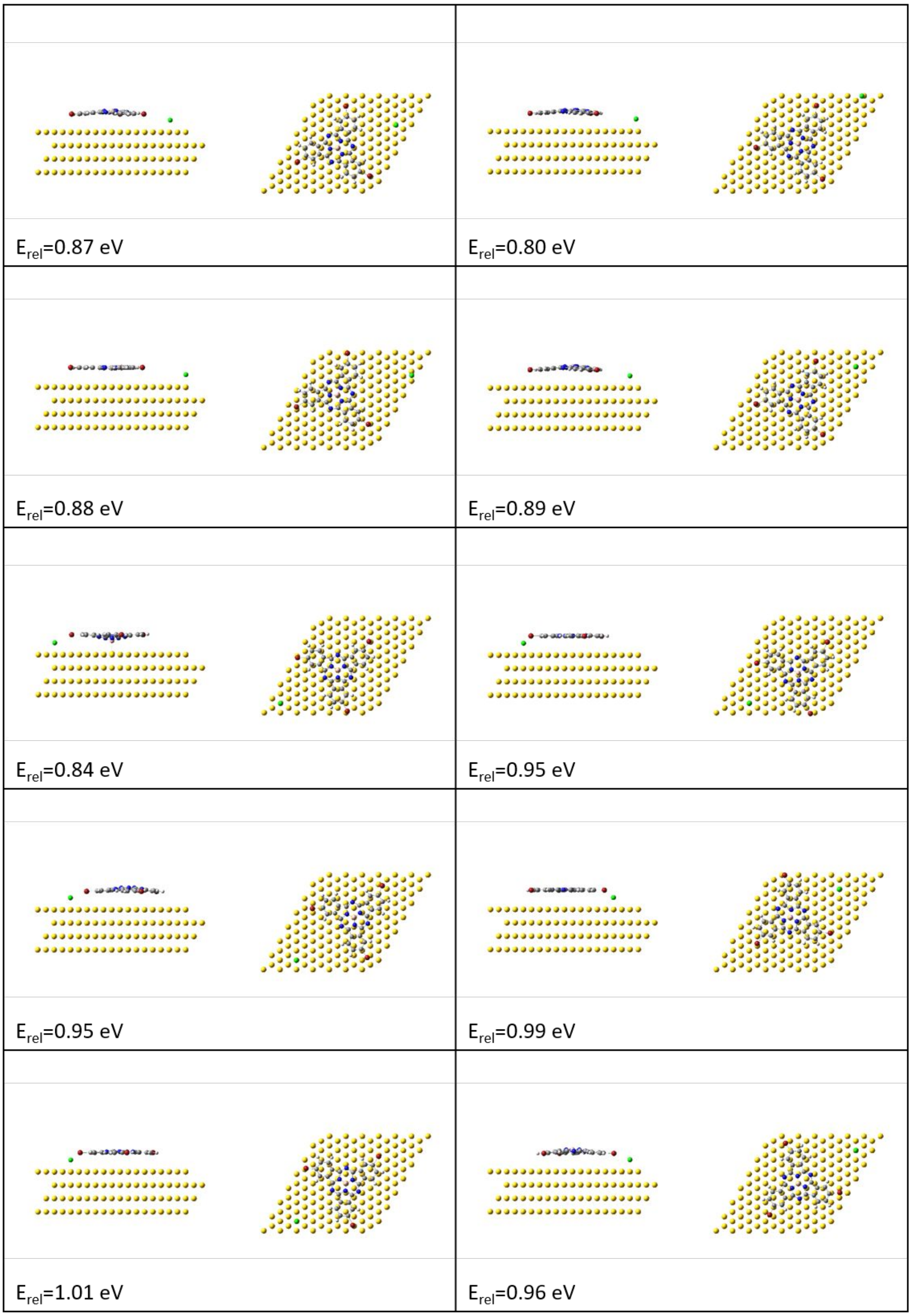

Figure S8.1-cont.

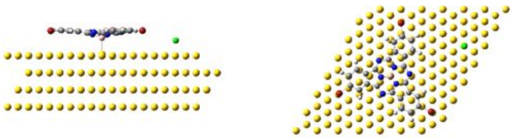	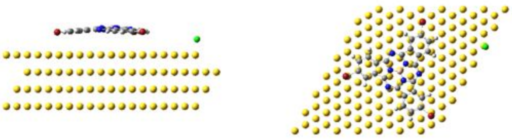
E_{rel}=0.81 eV	E_{rel}=0.79 eV
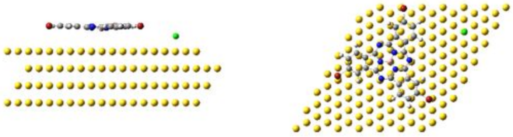	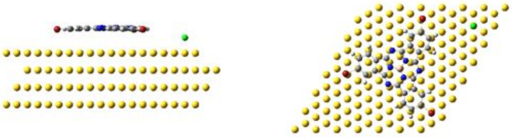
E_{rel}=0.99 eV	E_{rel}=0.89 eV
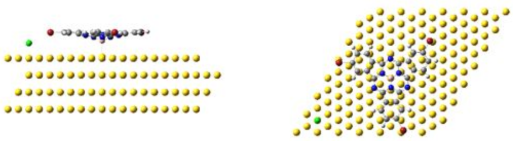	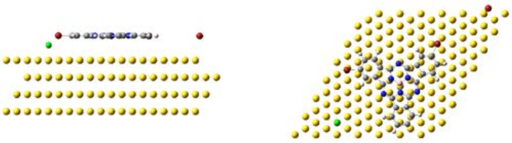
E_{rel}=0.84 eV	E_{rel}=0.96 eV
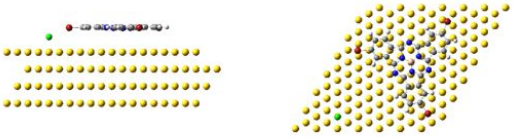	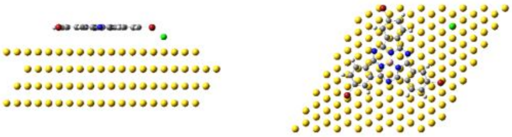
E_{rel}=0.94 eV	E_{rel}=0.99 eV
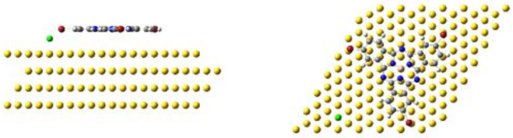	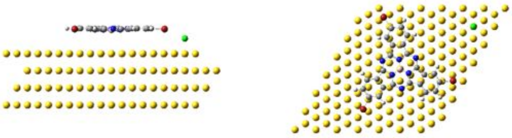
E_{rel}=1.00 eV	E_{rel}=0.95 eV

Figure S8.1-cont.

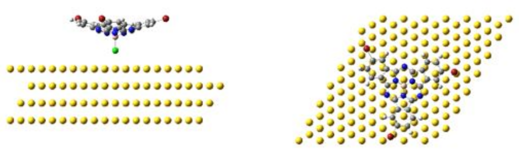	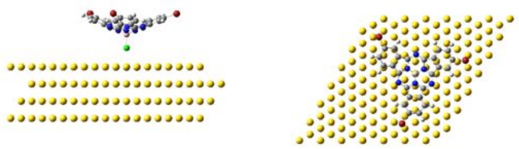
E_{rel}=2.11 eV	E_{rel}=2.11 eV
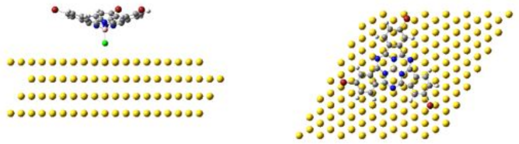	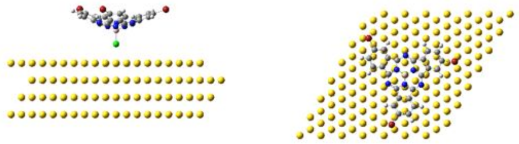
E_{rel}=2.10 eV	E_{rel}=2.11 eV
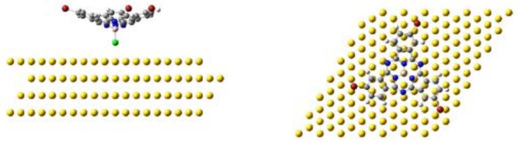	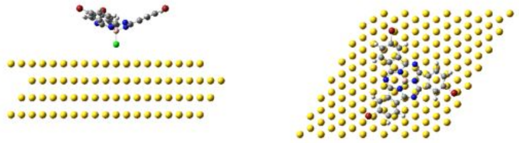
E_{rel}=2.11 eV	E_{rel}=2.12 eV
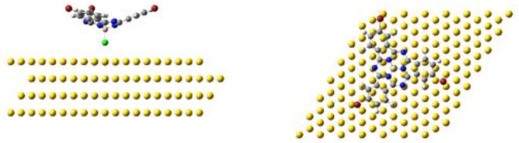	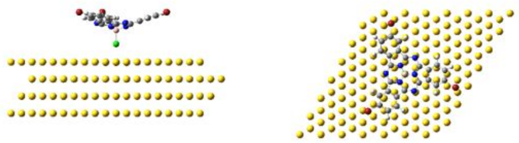
E_{rel}=2.11 eV	E_{rel}=2.11 eV

Figure S8.1-cont.

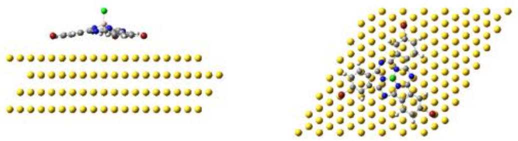 E_{rel}=0.00 eV	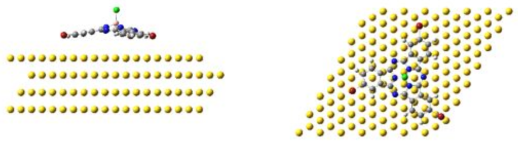 E_{rel}=0.19 eV
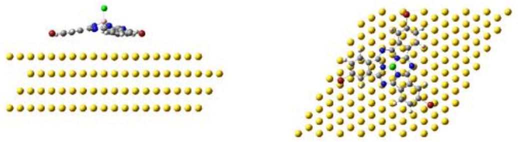 E_{rel}=0.22 eV	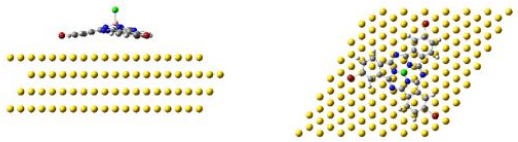 E_{rel}=0.01 eV
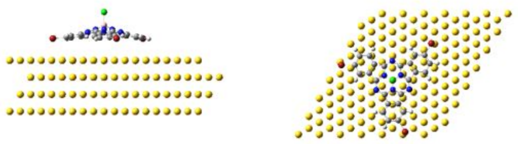 E_{rel}=0.10 eV	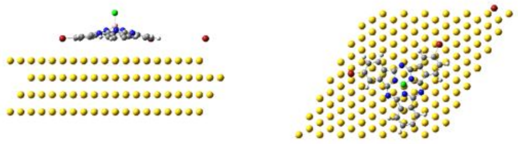 E_{rel}=0.09 eV
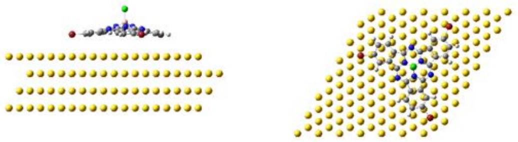 E_{rel}=0.08 eV	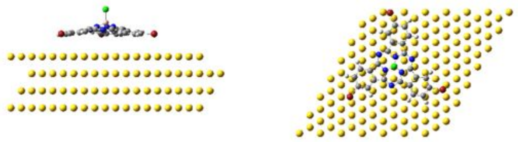 E_{rel}=0.15 eV
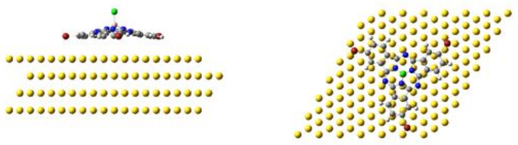 E_{rel}=0.15 eV	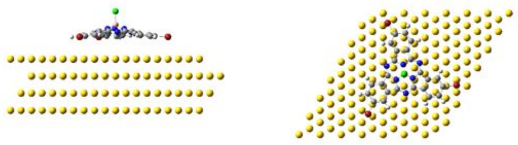 E_{rel}=0.08 eV

Figure S8.1-cont.

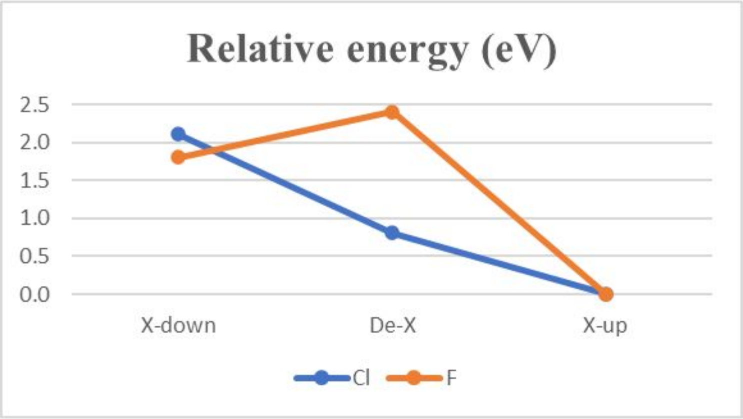

Figure S8.2. Relative energy comparison between chlorine (blue) and fluorine (red) axial ligands for the on-surface catalyzed axial dehalogenation on the Au(111) surface.

9. Supplementary Information references

[1] I. Sanchez-Molina, B. Grimm, R. M. Krick Calderon, C. G. Claessens, D. M. Guldi, T. Torres *J. Am. Chem. Soc.* 2013, ***135***, 10503.
[2] C. G. Claessens, T. Torres *Tetrahedron Lett.* 2000, ***41***, 6361.
[3] T. Yanai, D. P. Tew, N. C. Handy *Chemical Physics Letters* 2004*,* ***393***, 51.
[4] Gaussian 16, Revision C.01; Frisch, M. J.; Trucks, G. W.; Schlegel, H. B.; Scuseria, G. E.; Robb, M. A.; Cheeseman, J. R.; Scalmani, G.; Barone, V.; Petersson, G. A.; Nakatsuji, H.; Li, X.; Caricato, M.; Marenich, A. V.; Bloino, J.; Janesko, B. G.; Gomperts, R.; Mennucci, B.; Hratchian, H. P.; Ortiz, J. V.; Izmaylov, A. F.; Sonnenberg, J. L.; Williams-Young, D.; Ding, F.; Lipparini, F.; Egidi, F.; Goings, J.; Peng, B.; Petrone, A.; Henderson, T.; Ranasinghe, D.; Zakrzewski, V. G.; Gao, J.; Rega, N.; Zheng, G.; Liang, W.; Hada, M.; Ehara, M.; Toyota, K.; Fukuda, R.; Hasegawa, J.; Ishida, M.; Nakajima, T.; Honda, Y.; Kitao, O.; Nakai, H.; Vreven, T.; Throssell, K.; Montgomery, J. A., Jr.; Peralta, J. E.; Ogliaro, F.; Bearpark, M. J.; Heyd, J. J.; Brothers, E. N.; Kudin, K. N.; Staroverov, V. N.; Keith, T. A.; Kobayashi, R.; Normand, J.; Raghavachari, K.; Rendell, A. P.; Burant, J. C.; Iyengar, S. S.; Tomasi, J.; Cossi, M.; Millam, J. M.; Klene, M.; Adamo, C.; Cammi, R.; Ochterski, J. W.; Martin, R. L.; Morokuma, K.; Farkas, O.; Foresman, J. B.; Fox, D. J. Gaussian, Inc., Wallingford CT, 2016.
[5] G. Roman-Perez, J. M. Soler *Phys. Rev. Lett.* 2009*,* ***103***, 096102.
[6] a) G. Kresse, J. Furthmüller *Phys. Rev. B* 1996*,* ***54***, 11169-11186. b) G. Kresse, J. Furthmuller *Comput. Mater. Sci.* 1996*,* ***6***, 15-50.
